# Advances in Clinical Trials of Boron Neutron Capture Therapy

**DOI:** 10.34133/research.0988

**Published:** 2026-01-08

**Authors:** Xiaoling Li, Zhijie Liu, Kejun Liu, Chunhong Wang, Zhigang Liu, Xiao Xu

**Affiliations:** ^1^ Cancer Center, Dongguan Engineering Research Center for Innovative Boron Drugs and Novel Radioimmune Drugs, The Tenth Affiliated Hospital, Southern Medical University (Dongguan People’s Hospital), Southern Medical University, Dongguan 523059, China.; ^2^ Guangdong Engineering Research Center of Boron Neutron Therapy and Application in Malignant Tumors, The Tenth Affiliated Hospital, Southern Medical University (Dongguan People’s Hospital), Southern Medical University, Dongguan 523059, China.; ^3^ College of Chemistry and Molecular Engineering, Peking University, Beijing 100871, China.; ^4^ Department of Diagnostic Radiology, Yong Loo Lin School of Medicine, National University of Singapore, Singapore 119074, Singapore.

## Abstract

Boron neutron capture therapy (BNCT) is a targeted radiotherapy technique that enables dual targeting at the physico-biological level. Efficient neutron sources and specific boron carriers are essential for successful treatment. By exploiting the ^10^B(n,α)^7^Li nuclear reaction, the BNCT enables precise eradication of ^10^B-loaded tumor malignant cells while sparing adjacent healthy tissues, rendering it particularly advantageous for treating invasive or radioresistant recurrent tumors. Numerous clinical trials related to BNCT have focus on evaluating the safety and efficacy of treating glioblastoma, head and neck carcinoma, meningioma, malignant melanoma, and liver cancer. Preliminary studies have shown that BNCT treatment may extend the overall survival (OS) and improve the quality of life for patients with these cancers. Additionally, the scope of BNCT clinical trials has expanded to other tumor types, such as lung cancer, breast cancer, extramammary Paget’s disease, osteosarcoma, clear cell sarcoma, malignant peripheral nerve sheath tumor, angiosarcoma, thyroid cancer, recurrent chordoma, and gastrointestinal malignancies. This review provides a comprehensive overview of BNCT clinical trials, covering the therapeutic principles of BNCT and the current status and outcomes of clinical trials for various types of tumors. With advancements in neutron beam quality and the development of efficient, specific boron carriers, the therapeutic efficacy of BNCT is expected to be further enhanced, and its scope of application is anticipated to expand. Looking ahead, the BNCT is expected to be integrated with other tumor treatment modalities to augment local tumor control, thereby improving patients’ OS and quality of life.

## Introduction

Boron neutron capture therapy (BNCT) is a binary targeted radiotherapy modality. Compared with conventional radiotherapy, the BNCT offers higher targeting specificity, greater precision, and lower radiotoxicity. These characteristics make BNCT particularly suitable for treating malignant tumors with infiltrative growth and poorly defined boundaries, offering a novel therapeutic approach to improve the prognosis of these refractory tumors. The development of BNCT dates back to 1936, when Locher first proposed the theoretical basis for neutron capture therapy [1]. In 1951, American neurosurgeon Sweet [2] initiated the first clinical trial of BNCT for glioblastoma (GBM) at the Brookhaven Graphite Research Reactor, and the patient’s condition improved in the short term, although these patients eventually died of disease progression. As the results of subsequent BNCT clinical trials for GBM were unsatisfactory and pathological examinations revealed obvious radiation necrosis and various types of vascular lesions in some participants, the United States suspended the clinical trials of BNCT and turned to basic research on BNCT and the development of new boron-containing drugs. It was not until the emergence of second-generation boron-containing drugs, sodium borocaptate (BSH) and boronophenylalanine (BPA), that the BNCT-related clinical trials gradually began to be resumed worldwide and expanded from GBM to meningioma, melanoma, recurrent head and neck tumors, liver cancer, and other tumors [1,3].

Notably, the combination of BNCT with other therapeutic modalities has emerged as a research hotspot. Preclinical studies have demonstrated that BNCT combined with immune checkpoint inhibitors exhibits synergistic anticancer effects: Firstly, the DNA damage induced by BNCT can promote tumor antigen presentation; secondly, reprogramming the immune microenvironment may reverse the immunosuppressive state of tumors [4].

## The Basic Principle of BNCT

**Fig. 1.**
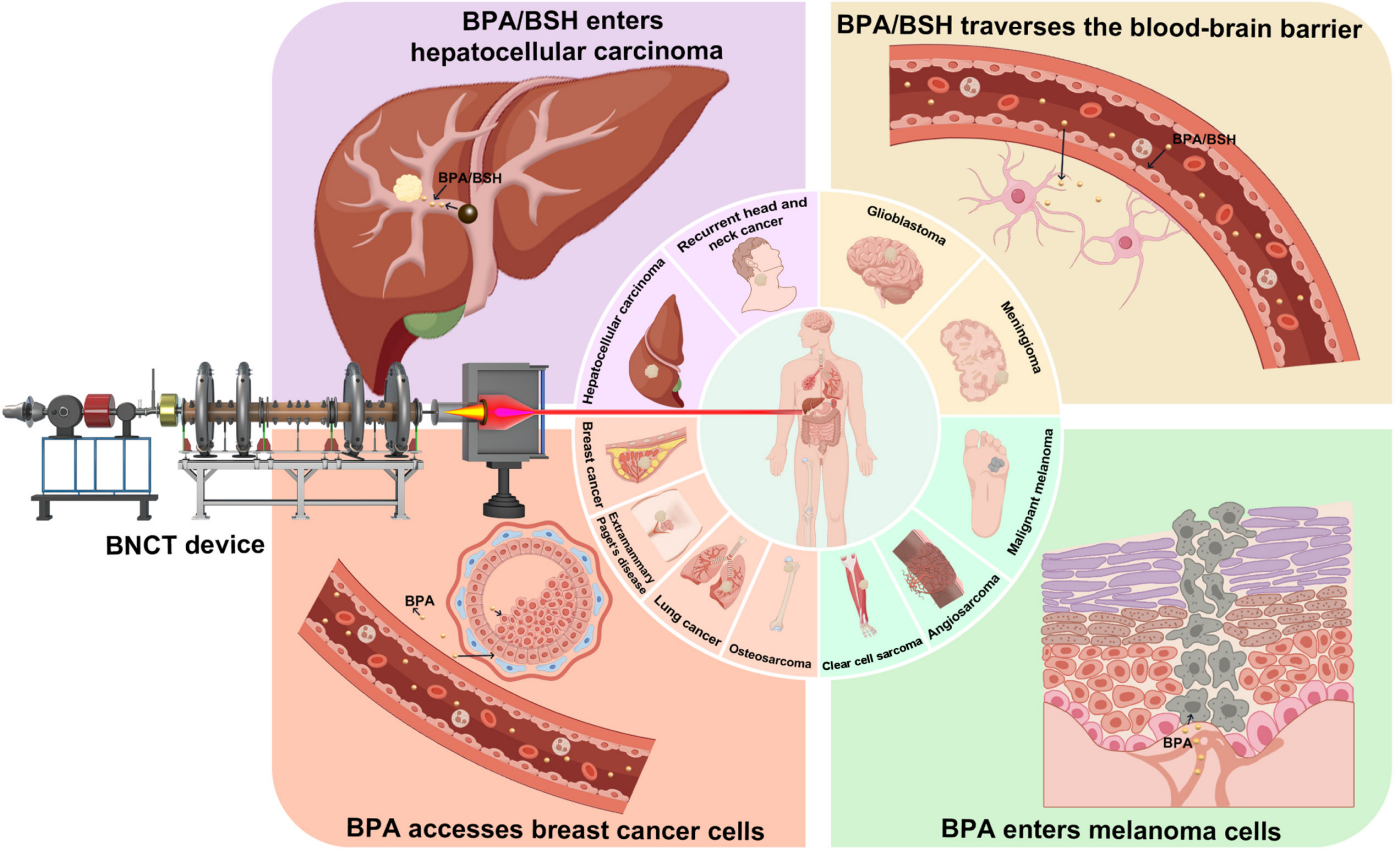
Types of tumors treated by BNCT.

The fundamental therapeutic principle of BNCT involves selectively accumulation of boron compounds, which exhibit a specific affinity for tumors, within tumor cells. Subsequently, tumor tissue is locally irradiated with epithermal or thermal neutron beams, inducing capture reactions between neutrons and ^10^B in tumor cells [5] (Fig. 1). This process leads to the fission of the unstable ^11^B isotope, resulting in the production of high-energy, short-range α particles and recoil lithium nuclei (^7^Li) [6,7]. These particles, characterized by their high relative biological effectiveness (RBE) and linear energy transfer (LET), are able to efficiently induce irreparable double-strand breaks in the DNA of tumor cells [8,9]. The short range (approximately 4.5 to 10 μm) of these particles ensures that they damage the target tumor cells (approximately 20 μm) while minimizing impact on surrounding normal cells [8,10]. The cytotoxic effect of the neutron capture reaction is primarily localized to tumor cells that have absorbed sufficient amounts of boron, sparing normal cells and tissues with low boron content.

Compared with conventional radiochemotherapy, the BNCT has several unique advantages, including the following: (a) The BNCT demonstrates tumor universality. The α particles generated by BNCT can not only eliminate tumor cells in both the proliferative and quiescent phases but also kill hypoxic tumor cells. This contrasts with traditional radiotherapy and chemotherapy, which primarily target rapidly dividing cells and are less effective against quiescent brain tumor stem cells and hypoxic tumor cells [11,12]. (b) The BNCT exhibits irreversibility. The lethal and potentially lethal damage caused by BNCT cannot be repaired by DNA. This makes it particularly effective against refractory tumors that can repair DNA damage following conventional chemotherapy and radiotherapy, thereby more effectively inhibiting tumor recurrence [13]. (c) The BNCT is a binary treatment modality. The neutron beam alone does not induce cell death. Only those cells that have absorbed the neutron capture agent and are subsequently irradiated by neutrons are damaged. The drug itself has low toxicity and minimal side effects, thereby providing the targeting specificity of local irradiation [14].

## The Composition of BNCT

The key components of BNCT primarily consist of the neutron source, neutron beam, boron delivery agent, and real-time measurement system for boron concentration and dose [1,15]. Neutron sources can be classified into reactor-based neutron sources and accelerator-based neutron sources [6,14]. Due to the high cost, large footprint, high maintenance expenses, and nuclear waste generation associated with reactor-based neutron sources, many reactors have ceased BNCT trials and are no longer accessible. Currently, only a few reactors are still available for BNCT, including the Kyoto University Research Reactor (KURR), the Massachusetts Institute of Technology Reactor (MITR), the University of Missouri Research Reactor, and the in-hospital neutron irradiator (IHNI) based on the improved miniature neutron source reactor in China [14,16]. In light of these circumstances, accelerator-based neutron sources, which offer enhanced safety and simplified operation, have been developed to generate neutron beams for irradiation therapy. At present, Japan has 4 hospitals equipped with accelerator-based BNCT facilities (AB-BNCT): the NeuCure system at Minato-Mirai Hospital, the NeuCure at Kansai BNCT Medical Center, the CICS-1 at the National Cancer Center Hospital, and the beryllium-target-based AB-BNCT at the University of Tsukuba Hospital [17]. Additionally, in Finland, a NuBeam accelerator-based neutron source with a lithium target was constructed at the Helsinki University Hospital in 2018 and was put into service in 2024 [14,18]. In recent years, China has also initiated the construction of AB-BNCT devices, such as the NeuPex AB-BNCT system at Xiamen Hong-ai Hospital and the D-BNCT 02 device at Dongguan People’s Hospital [6,16].

Neutrons can be classified into thermal neutrons, epithermal neutrons, and fast neutrons [6]. Thermal neutrons, characterized by their low energy, exhibit limited penetration through tissues, thereby restricting their application primarily to the treatment of superficial tumors. In contrast, epithermal neutrons, which are decelerated by the surface tissues, are thermalized to thermal neutrons by the time the neutron beam reaches the tumor region, rendering them more suitable for the treatment of deep-seated tumors.

Boron-containing drugs encompass boric acid and its derivatives, BPA, BSH, and boron carriers that integrate various targeting molecules with boron compounds, exhibiting enhanced targeting properties [6,8]. The BNCT requires boron drugs to meet the following criteria: low toxicity in normal tissues, minimal uptake by normal tissues, high tumor targeting ability, a tumor cell ^10^B concentration of 20 μg-^10^B/g, and a boron concentration ratio of tumor to normal tissue and tumor to blood greater than 3:5. The first generation of boron drug delivery agents, such as boric acid and its derivatives, were initially used but are now rarely employed due to their low selectivity and tumor specificity [1].

The second generation of boron drugs, including BPA and BSH, are currently widely used in BNCT research and clinical trials and are the only boron drugs approved by the U.S. Food and Drug Administration for BNCT clinical trials [6]. BPA, an analog of phenylalanine, can enter the brain via the l-amino acid transport system in the blood–brain barrier, thereby enhancing the uptake of BPA by glioma cells [19,20]. BPA is the most widely used boron compound in clinical trials. However, with its relatively low solubility, it is typically complexed with fructose to form an infusion solution to enhance its water solubility [1,3,21]. BSH is a polyhedral sulfhydryl boron molecule that possesses a high boron-carrying capacity, as each molecule incorporates 12 ^10^B atoms (Na₂B₁₂H₁₁SH) [22,23]. Therapeutic studies of BSH have primarily targeted malignant brain tumors, particularly in those with disrupted blood–brain barrier function, where BSH can enter and accumulate within the tumor region. However, BSH lacks a specific mechanism for cellular uptake and typically enters the brain when the blood–brain barrier is compromised [3,21]. The tumor targeting ability of these 2 drugs is moderate, and they are rapidly cleared from the bloodstream after entering the human body, which means that they cannot fully meet the current demands for boron drugs.

In response to this issue, the third generation of boron carriers composed of targeting molecules and boron compounds has been developed [8,24]. Small-molecule boron carriers include boronated amino acids [25,26], boronated peptides [27], boronated porphyrins [28], boron-containing nucleosides [29], and boronated DNA intercalators [30]. Boron-conjugated bioconjugates include boronated liposomes [31], boronated monoclonal antibodies [32], boron-containing nanomaterials [33,34], and boron nitride nanotubes [35,36]. In addition, receptors that are highly expressed on the surface of tumor cells, such as epidermal growth factor receptor [37], vascular endothelial growth factor receptor [38], and HER2 receptor [39], can also be conjugated with boron compounds to achieve targeted drug delivery.

Compared with BPA and BSH, boron compounds conjugated with targeting antibodies or ligands exhibit higher tumor targeting ability and enhance the effective uptake of drugs by tumor cells. Boronated porphyrin derivatives and their analogs have low toxicity and good tumor affinity. Moreover, boronated liposomes and nanomaterials have higher boron-carrying capacities, which can increase the intracellular ^10^B content. However, these novel boron drugs are still in the experimental research stage. The receptor expression differences among different patients or within the same tumor can easily lead to unstable drug uptake, affecting the expected therapeutic effect. Additionally, some formulations, such as nanodrugs and antibody conjugates, are large in size, have poor penetration ability, and are difficult to cross the blood–brain barrier, which limits their application. Both small-molecule boron compounds and boron-conjugated bioconjugates exhibit certain biological activities and pharmacological effects, laying a solid foundation for their application in BNCT. Numerous books and review articles have provided detailed discussions not only on boron chemistry but also on the development of boron-containing compounds as BNCT agents, delving into their molecular design, targeted delivery, pharmacological mechanisms, and preclinical applications [1,3,15,21,24,40–46]. Building upon this foundational knowledge, marked progress has been made in this field. Taking the work of Zhu and colleagues [47–50] as an example, the team has conducted systematic investigations into the properties of boron clusters and their medical applications. They have not only thoroughly analyzed the physicochemical characteristics of boron clusters but also developed a series of novel boron-containing materials and bioconjugates. These novel systems demonstrate excellent targeting performance in biomedical applications, highlighting their potential research value and promising prospects for practical use. This review, however, primarily focuses on the progress of BNCT clinical trials.

## The Clinical Study of BNCT in the Treatment of Tumors

**Fig. 2.**
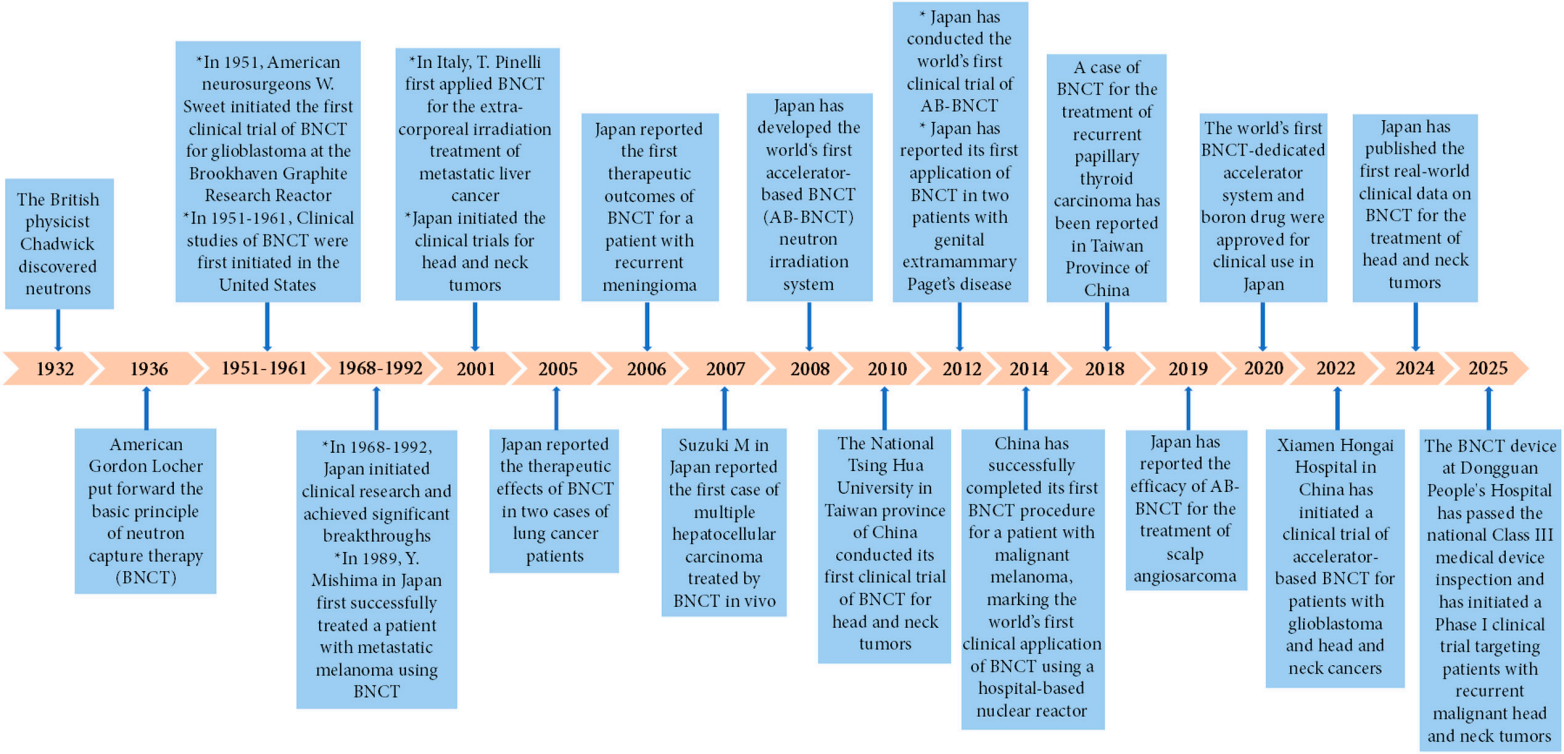
The developmental history of BNCT.

The BNCT is characterized by its high targeting ability, tumor specificity, and minimal damage to surrounding tissues. It is suitable for treating invasive, multifocal, recurrent, and radioresistant tumors. Since the initial clinical trial of malignant brain tumors conducted by Sweet et al. in the Brookhaven graphite reactor in 1951, the clinical applications of BNCT have progressively extended to over 10 types of difficult-to-treat tumors, such as gliomas, recurrent head and neck tumors, melanomas, and liver cancers (Fig. 2). The ongoing clinical trials also focus on other tumor types, including lung cancer, breast cancer, extramammary Paget’s disease, osteosarcoma, clear cell sarcoma, malignant peripheral nerve sheath tumor, angiosarcoma, thyroid cancer, recurrent chordoma, and gastrointestinal malignancies.

### Clinical trials for GBM

GBM, a tumor originating from neuroglial cells, is the most common primary intracranial tumor, accounting for approximately 80% of all malignant brain tumors [51]. The World Health Organization (WHO) classifies GBMs into grades 1 to 4, with grades 1 and 2 being low-grade gliomas and grades 3 and 4 being high-grade gliomas [52]. High-grade gliomas are highly malignant, with a median overall survival (OS) of less than 2 years and a 5-year OS of only about 10% [51]. The conventional treatment modalities for GBM currently include surgery, chemotherapy, and radiotherapy, with surgery as the first choice, followed by postoperative chemoradiotherapy. The Stupp regimen, which combines postoperative radiotherapy with temozolomide, is the standard adjuvant radiotherapy protocol for gliomas [53]. However, the prognosis for most patients still remains poor. Therefore, improving patient prognosis and extending survival are still key issues that need to be addressed in current GBM research.

It is worth noting that although surgical resection is the preferred treatment for gliomas, it has several drawbacks, such as high invasiveness, high risk of postoperative complications, and negative impact on patients’ long-term quality of life. In addition, the extent and completeness of surgical resection are limited by various factors, making it difficult to completely eliminate microscopic tumor cells, thereby affecting patients’ prognosis. In this context, as a noninvasive and precise treatment method, the BNCT offers a new approach for the treatment of gliomas. Numerous clinical trials investigating BNCT for GBM have been conducted (Table 1). Between 1951 and 1953, the first patients with malignant gliomas were treated with BNCT at the Brookhaven Medical Research Reactor (BMRR) [2,54]. Sweet et al. [55] conducted BNCT on 17 patients (16 with GBM and 1 with medulloblastoma) at the MITR, achieving a median survival of 5.7 months in this cohort. Additionally, Asbury et al. [56] conducted a neuropathological study on 14 patients who had received BNCT at the MIT nuclear reactor between 1959 and 1961, finding that 9 patients exhibited extensive radiation necrosis and various types of vascular lesions. The clinical trials used boric acid and its derivatives as boron drugs, which had poor targetability to tumor cells and failed to specifically accumulate within tumor. Consequently, this resulted in high levels of ^10^B in the blood and normal tissues. Following neutron irradiation, normal tissues suffered marked radiation damage. Moreover, the weak penetration ability of thermal neutrons resulted in insufficient doses reaching the tumor tissue, failing to achieve targeted tumor cell destruction. Due to these less-than-ideal outcomes, clinical trials of BNCT in the United States were suspended.

**Table 1.** Clinical trials of glioblastoma

Author	Time	Country/region	Boron drug	Neutron source	Neutron type	Tumor type	Outcomes	Number of cases
Sweet et al. [2]	1951	USA	Boric acid	BMRR	Thermal	GBM	Died	1
Sweet et al. [55]	1961	USA	Para-boron phenylalanine	MITR	Thermal	GBM/medulloblastoma	MST: 5.7 months	16/1
Hatanaka et al. [57]	1968	Japan	BSH	HTR, JRR-3, MTII, KURR, JRR-2	Epithermal	GBM/meningioma	MST: 640 d	120/29
Chadha et al. [58]	1994–1995	USA	BPA	BMRR	Thermal	GBM	Median time to PD: 6 months	10
Chanana et al. [59]	1996–1998	USA	BPA	BMRR	Epithermal	GBM	Tumor progression: 30 patients	38
Busse et al. [60]	1996–1999	USA	BPA	MITR	Epithermal	GBM/melanoma	MST: 13 months; CR: 2 patients	20/2
Sauerwein et al. [61]	1997	Holland	BSH	HFR	Epithermal	GBM	MST: 8.4 months	10
Nakagawa et al. [64]	1998–2001	Japan	BSH	JRR-4	Thermal and epithermal	GBM	Tumor-free survival: 5 patients	10
Kageji et al. [65]	1998–2004	Japan	BSH	KURR	Thermal and epithermal	GBM	The first group (with GTV dose of 15 Gy): MST (15.3 months); 1-year OS rate (66.7%); the second group (with CTV dose of 18 Gy): MST (19.5 months); 1-year OS rate (60.6%); 2-year OS rate (37.9%)	19
Yamamoto et al. [71]	1998–2007	Japan	BPA, BSH	JRR-4	Epithermal	GBM	MST: 25.7 months; TTP: 11.9 months; 1-year OS rate: 80.0%; 2-year OS rate: 53.3%	15
Kageji et al. [83]	1998–2005	Japan	BPA, BSH	KURR	Epithermal	GBM	Overall MST: 19.5 months; 2-year OS rate: 31.8%; 3-year OS rate: 22.7%; 5-year OS rate: 9.1%	23
Joensuu et al. [66]	1999–2003	Finland	BPA	FiR 1	Epithermal	GBM	OS rate: 61%	21
Diaz et al. [62,63]	1994–1999	USA	BPA	BMRR	Epithermal	GBM	MST: 12.8 months; median time to progression: 28.4 weeks	53
Miyatake et al. [67]	2002–2003	Japan	BPA, BSH	KURR	Epithermal	GBM	10 died	13
Miyatake et al. [68]	2002–2007	Japan	BPA, BSH	KURR	Epithermal	GBM	MST: 10.8 months	22
Kawabata et al. [70]	2002–2006	Japan	BPA, BSH	KURR	Epithermal	GBM	MST: 15.6 months	21
Nakai et al. [82]	2005–2007	Japan	BPA, BSH	JRR-4	Epithermal	GBM	Median OS: BNCT (+) group (24.4months) BNCT (-) group (14.9 months)	33
Kankaanranta et al. [72]	2007–2009	Finland	BPA	FiR 1	Epithermal	GBM/anaplastic astrocytoma	Median time to progression: 3 months median MST: 7 months	20/2
Miyatake et al. [197]	2013–2018	Japan	BPA	KURR	Epithermal	Recurrent glioblastoma	The first cohort (7 patients): median OS (15.1 months); median PFS (5.4 months)	13
Furuse et al. [90]	2013–2019	Japan	BPA	KURR	Epithermal	GBM	Primary GBM: 1-year OS rate (63.5%); median OS (21.4 months); median PFS (8.3 months); 1-year OS rate (81.8%) non-primary GBM: median OS (73.6 months); median PFS (15.6 months)	25
Kawabata et al. [76,77]	2016–2018	Japan	BPA	AB-BNCT	Epithermal	GBM	Median OS: 18.9 months; 1-year OS rate: 79.2%;2-year OS rate: 33.3%;3-year OS rate: 20.8%	27
Lan et al. [73]	2017	Taiwan province of China	BPA	THOR	Epithermal	GBM	Tumor recurrence	3
Chen et al. [74]	2017–2019	Taiwan province of China	BPA	THOR	Epithermal	GBM	ORR: 50%; disease control rate: 85.3%; OS: 7.25 months relapse-free survival times: 4.18 months	34
Huang et al. [79]	2019–2022	Taiwan province of China	BPA	THOR	Epithermal	Recurrent diffuse midline glioma	OS: 6.39 months recurrence-free survival times: 4.35 months	6
Shimizu et al. [91]	2023	Japan	BPA	Proton Medical Research Center	Epithermal	Recurrent glioblastoma	Died after 64 months	1

BMRR, Brookhaven Medical Research Reactor; MITR, Massachusetts Institute of Technology Reactor; HTR, Hitachi Training Reactor; JRR-3, Reactor of Japan Atomic Energy Research Institute; MTII, Reactor of Musashi Institute of Technology; KURR, Kyoto University Research Reactor; JRR-2, Reactor of Japan Atomic Energy Research Institute; HFR, High Flux Reactor at Petten; JRR-4, Japan Research Reactor No. 4; FiR 1, Reactor in Espoo, Finland; GBM, glioblastoma; MST, median survival time; PD, progressive disease; CR, complete response; OS, overall survival. AB-BNCT, accelerator-based BNCT facilities; THOR, the Tsing Hua Open-pool Reactor; GTV, gross tumor volume; CTV, clinical target volume; TTP, time to progression; PFS, progression-free survival; ORR, objective response rate

**Fig. 3.**
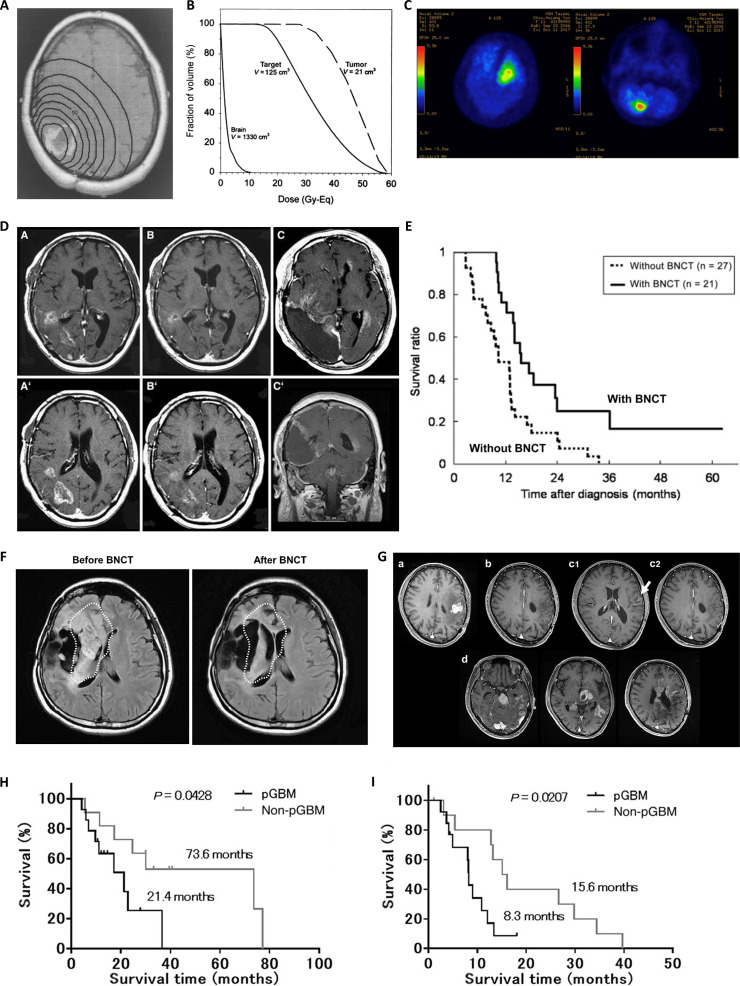
(A) Isodose curves for brain tumors [58]. (B) Dose–volume histogram for the tumor, target volume (tumor + 2 cm), and normal brain [58]. (C) The pre-BNCT ^18^F-BPA PET imaging revealed T/N ratios of 3.58 in the left frontal lobe and 4.58 in the right cerebellum [73]. (D) Representative MR images of BNCT treatment [68]: tumor location before BNCT (A and A′), marked reduction in the lesion 48 h post-BNCT (B and B′), and cerebrospinal fluid dissemination at 7 months post-BNCT (C and C′). (E) Comparison of Kaplan–Meier survival curves for BNCT treatment and historical treatments (non-BNCT) at the same institution [81]. (F) MRI before and 3 months after BNCT. Substantial reduction in tumor volume, representing a PR [74]. (G) Typical MR images of a GBM patient with a longer survival period during follow-up [90]: first recurrence 8.8 months post-surgery (a), tumor reduction and CR 3 months after BNCT (b), tumor recurrence (c1, white arrow) and control of the targeted original tumor volume 8.2 months after BNCT (c2), and death due to cerebrospinal fluid dissemination 36.7 months after BNCT (d). (H and I) Kaplan–Meier estimated PFS and OS for patients with recurrent malignant gliomas after BNCT [90]: (H) OS and (I) PFS.

In 1967, Hatanaka utilized the boron carrier BSH synthesized by Soloway to recommence BNCT experiments [20]. Between 1968 and 1992, Hatanaka’s team treated 149 patients, including 120 glioma patients, using 5 reactors in Japan, and conducting 164 craniotomies for BNCT. The overall effective rate for glioma patients was 64%, with median survival times (MST) of 640 d for GBM, 1,811 d for anaplastic astrocytoma, and 1,669 d for low-grade astrocytoma; 6 patients survived for over 10 years [57]. From September 1994 to July 1995, 10 patients underwent neutron beam irradiation at the BMRR. These patients received neutron irradiation after BPA infusion, and radiation dose maps were created (Fig. 3A and B); the median time to progressive disease (PD) after BNCT was 6 months [58]. Chanana et al. [59] conducted a phase I/II clinical trial of BNCT for 38 GBM patients who had undergone subtotal or gross total resection. After intravenous infusion of BPA-fructose, the patients received neutron irradiation. Post-BNCT, 30 patients experienced tumor progression and 25 died. Radiation-induced damage to normal brain tissue was observed in pathological examinations. Harvard-MIT carried out a phase I clinical trial of neutron capture therapy for intracranial diseases from July 1996 to May 1999. A total of 22 participants ultimately received irradiation treatment. Two achieved complete response (CR), and 13 had marked tumor shrinkage after neutron capture therapy. The overall MST for all participants was 13 months [60]. The first European clinical trial for brain glioma began in 1997. Ten GBM patients received BNCT. The MST post-BNCT was 8.4 months. At the time of analysis, 2 treated patients were still alive but experienced tumor recurrence [61]. Diaz et al. [62,63] conducted a retrospective analysis of a phase I/II dose-escalation study at the BMRR, enrolling a total of 53 glioma patients. Among them, 26 participants received treatment with 1 irradiation field, 17 with 2 fields, and 10 with 3 fields. The median time to progression (TTP) for all patient post-treatment was 28.4 weeks, and the median OS was 12.8 months. The median OS following BNCT with single-field, dual-field, and triple-field treatments was 14.8, 12.1, and 11.9 months, respectively. At the time of analysis, 4 patients were still alive, and 3 had been followed up for over 2 years without recurrence. No BPA-fructose-related toxicities were observed during the treatment process. All patients who received 3 irradiation fields experienced acute or subacute functional neurotoxicity. In contrast, the incidence of neurotoxicity was lower among those treated with 1 or 2 fields, suggesting that an increased radiation dose to the brain tissue may lead to a higher likelihood of side effects. Japan implemented a new BNCT treatment protocol from 1998 to 2001. Patients were intravenously infused with BSH and subsequently irradiated with a mixed beam of thermal and epithermal neutrons. A total of 10 glioma patients were treated. Two patients died due to tumor recurrence, and 3 died due to tumor dissemination. However, pathological examinations revealed no recurrence around the primary tumor site. The remaining 5 patients were still alive and tumor-free at the time of the report [64]. Kageji et al. [65] conducted a study applying BNCT using a mixed epithermal and thermal neutron beam to treat patients with malignant glioma. A total of 19 patients were included in the study. Eight patients received the first treatment regimen with a gross tumor volume (GTV) dose of 15 Gy, while 11 patients received the second treatment regimen with a clinical target volume (CTV) dose of 18 Gy. In the first group, the estimated MST for the 6 evaluable patients was 15.3 months, with a 1-year OS rate of 66.7%. No patient survived beyond 2 years. In the second group, the median OS was 19.5 months, with 1- and 2-year OS rates of 60.6% and 37.9%, respectively. Two patients with GBM survived for over 4 years after diagnosis. The average follow-up period was 21 months. At the time of analysis, 14 patients had died, with the longest survival reaching 52 months. Finland conducted 2 clinical trials on BNCT between 1999 and 2003. From 1999 to 2001, 18 patients treated with BNCT had an OS rate of 61%. Between 2001 and 2003, 3 patients were treated with BNCT, and their symptoms were alleviated within 3 months after BNCT [66]. Additionally, Miyatake et al. [67] conducted a BNCT trial using BPA and BSH as boron delivery agents for the first time, from January 2002 to December 2003. They treated a total of 13 patients with GBM, all of whom received irradiation at the KURR in Japan. Magnetic resonance imaging (MRI) and ^18^F-BPA positron emission tomography (PET) scans before BNCT showed high BPA uptake in tumors. One week after irradiation, the average tumor volume reduction was 46.4% for all patients. During the follow-up period, the average tumor volume reduction was 58.5%. Histological staining of tumor tissue sections revealed substantial necrosis and a decrease in the Ki-67 ratio. However, among the 13 patients, 10 ultimately died. Moreover, between 2002 and 2007, the team led by Miyatake et al. [68] treated a total of 22 patients using a combination of BPA and BSH therapy. The MST after BNCT for all patients was 10.8 months, with an MST of 9.6 months for recurrent patients. Some patients had a positive response to the treatment, with MRI scans taken 48 h after treatment showing a reduction in tumor volume (Fig. 3D). Kuroiwa et al. [69] also reported the treatment outcome of a recurrent GBM patient who underwent modified BNCT at the KURR. The patient received a combination of BPA and BSH as boron delivery agents. Forty-eight hours after BNCT, MRI showed a 70% reduction in contrast-enhanced lesions and a decrease in mass effect. The patient’s condition remained stable for 6 months following treatment. Since then, clinical studies have increasingly used a combination of BPA and BSH to enhance the efficacy of BNCT. Additionally, Kawabata et al. [70] conducted neutron capture therapy using a combination of BSH and BPA at the KURR. The study found that the MST for patients treated with BNCT combined with postoperative x-ray radiotherapy was longer than that for patients treated with BNCT alone. However, the difference between the 2 groups was not statistically significant. Yamamoto et al. [71] treated 15 newly diagnosed GBM patients with neutron irradiation at the Japan Research Reactor No. 4 (JRR-4). Seven patients received intraoperative neutron capture therapy after BSH infusion, while 8 patients received extracorporeal beam neutron capture therapy with a combination of BPA and BSH. The MST and TTP for all patients were 25.7 and 11.9 months, respectively. The 1- and 2-year survival rates were 80.0% and 53.3%, respectively, preliminarily indicating that BNCT can improve short-term survival in newly diagnosed GBM patients. Kankaanranta et al. [72] recruited 22 glioma patients, with a median time to disease progression of 3 months and a median overall MST of 7 months.

In 2017, Lan et al. [73] from Taipei Veterans General Hospital treated 3 patients with recurrent glioma using BNCT at the Tsing Hua Open-pool Reactor (THOR) in Taiwan province of China. Pretreatment ^18^F-BPA PET imaging showed high uptake of BPA by the tumors (Fig. 3C). All patients showed tumor regression within 3 months after BNCT. However, the 3 patients experienced tumor recurrence at 18, 11, and 4 months post-BNCT, respectively. Chen et al. [74] from the National Yang Ming Chiao Tung University conducted BNCT trials at THOR from 2017 to 2019, treating a total of 34 patients. The objective response rate (ORR) was 50%, and the disease control rate was 85.3%. Follow-up MRI at 3 months post-treatment in some patients showed reduced tumor volume (Fig. 3F). The study found that patients with a tumor/normal tissue boron concentration ratio (T/N) ≥ 4, tumor volume < 20 ml, average tumor dose ≥ 25 Gy-E, MIB-1 ≤ 40, and lower Recursive Partitioning Analysis classification had better outcomes from BNCT. Lan et al. [75] reported the outcome of BNCT salvage treatment for a patient with recurrent GBM. The patient had a left parietal GBM and had previously undergone surgery and postoperative chemoradiotherapy combined with temozolomide. However, recurrence occurred 6 months later. After evaluation, the patient was referred for BNCT treatment. Two weeks after treatment, MRI showed nearly achieving CR, reduced perifocal edema, and marked improvement in right-sided limb weakness. Follow-up cranial MRI 3 months later still showed stable disease (SD) without recurrence. Between 2016 and 2018, Kawabata et al. [76] treated a total of 27 patients with recurrent glioma using AB-BNCT after BPA infusion. The 1-year OS rate and median OS for recurrent GBM cases were 79.2% and 18.9 months, respectively, indicating that AB-BNCT was more effective in controlling tumors than standard therapies. The long-term follow-up of 24 patients showed a median OS of 19.2 months, with 2- and 3-year survival rates of 33.3% and 20.8%, respectively. These results further support the preliminary evidence for BNCT’s efficacy in prolonging OS [77]. Chen et al. [78] conducted salvage BNCT treatments for 5 pediatric patients with recurrent brain tumors from March 2017 to January 2020, performing a total of 9 treatments (involving 10 sites). Within 3 months after BNCT, follow-up MR images demonstrated that among the 10 irradiated sites, 1 site nearly achieved CR, 3 sites showed partial response (PR), 4 sites had SD, and 2 sites exhibited disease progression. The median follow-up time post-BNCT was 5.3 months, with a median progression-free survival (PFS) of 3.8 months. Tumor responses were observed in all patients. However, 4 patients died due to subsequent disease progression. From September 2019 to July 2022, Taipei Veterans General Hospital and National Yang Ming Chiao Tung University treated 6 children with recurrent diffuse midline glioma. The average age was 9.7 years (range, 6 to 17 years). All patients received 2 BNCT treatments combined with bevacizumab therapy. Among these patients, 3 achieved PR and 3 had SD. The OS and recurrence-free survival times were 6.39 and 4.35 months, respectively. Only one patient experienced brain necrosis, while the remaining patients did not suffer from severe adverse events [79,80].

Additionally, Kawabata et al. [81] compared the efficacy of BNCT with conventional treatments, treating 21 newly diagnosed GBM patients with neutron irradiation at the JRR-4 reactor. The results showed that the MST for patients treated with BNCT was longer than that for those treated with conventional treatments (Fig. 3E). Nakai et al. [82] also compared the efficacy of BNCT and conventional treatments, finding that in high-risk patients (Class V from the Recursive Partitioning Analysis), the median OS in the neutron capture therapy (+) group tended to be better than that in the conventional treatment group. Kageji et al. [83] compared the MST and survival rates between intra-operative BNCT (IO-BNCT) and non-operative BNCT (NO-BNCT). The overall MST for all participants was 19.5 months, with 2-, 3-, and 5-year OS rates of 31.8%, 22.7%, and 9.1%, respectively. The MST and OS rates for NO-BNCT were superior to those for IO-BNCT. The team further investigated the impact of radiation dose on the survival and pathological changes of subjects, finding that a higher radiation dose was beneficial for tumor control and survival extension [84]. Taipei Veterans General Hospital conducted a retrospective analysis of the correlation between T/N ratios in ^18^F-BPA-PET scans and BNCT outcomes in malignant brain tumors. A total of 116 patients were evenly divided into 2 groups. The first group received BNCT treatment following ^18^F-BPA-PET scans, while the second group underwent ^18^F-BPA-PET scans only. Forty-four patients (75.9%) in the first group and 26 patients (44.8%) in the second group showed T/N ratios greater than 2.5. The median OS was 8.5 months for the first group and 6.0 months for the second group. Among all patients, there was no meaningful difference in OS rates concerning different T/N ratios. In the first group, 55 patients who received BNCT treatment were assessed for response. It was found that, in patients with CR (12.7%) and ORR (30.9%), those with T/N ratios greater than 2.5 had markedly higher OS than those with T/N ratios less than 2.5 [85]. Kamano et al. [86] reported a case of a patient with diffuse astrocytoma who survived for 32 years after BNCT. An MRI performed 35 d after the initial BNCT showed no residual tumor. Six months post-BNCT, the patient was able to return to work. However, the patient was readmitted 7 years later due to gradually worsening neurological conditions and underwent a second surgical treatment. The condition stabilized, and follow-up MRIs at 11 and 12 years post-second surgery showed improvement in the lesion with a reduction in the size of the residual cyst. During the 20-year follow-up period post-second surgery, no notable changes were observed in MR images.

Long-term survivors received higher neutron radiation doses, but this also led to radiological damage such as brain atrophy, cerebral white matter lesions, and neuronal injury, as well as pseudoprogression following BNCT treatment [87]. Studies have shown that bevacizumab can control radiological damage and pseudoprogression. Miyatake’s team [88] reported 2 cases of pseudoprogression in glioma patients following BNCT that were successfully controlled with bevacizumab. Additionally, Miyatake and colleagues [89] designed 2 cohorts to investigate the effects of BNCT combined with early bevacizumab treatment for recurrent malignant gliomas. From June 2013 to May 2014, 7 patients were enrolled in the first cohort, and 6 patients were treated from August to December 2017. Both groups received early bevacizumab treatment after BNCT. All patients were followed up until April 2018. At the time of analysis, the median OS and PFS for the first group were 15.1 and 5.4 months, respectively, while the second group had not yet reached median OS and PFS due to the short follow-up period. No symptomatic worsening of radiation-induced brain necrosis was observed during the treatment process.

Furuse et al. [90] conducted BNCT treatments for 25 patients at KURR from June 2013 to February 2019. The results indicated that BNCT combined with bevacizumab treatment provided longer OS and PFS for recurrent malignant gliomas, with some patients showing good treatment response (Fig. 3G to I). Shimizu et al. [91] treated a patient with recurrent GBM after surgery. Pseudoprogression occurred 1 month post-BNCT, but the patient was able to maintain daily living activities at home for 5 years after bevacizumab control. The patient died 64 months post-BNCT due to worsening general condition.

The above multiple clinical studies have demonstrated that BNCT has shown certain therapeutic efficacy in brain gliomas, particularly in recurrent GBM, including tumor reduction, symptom relief, and extended short-term survival. However, some long-term survivors have experienced adverse effects, including radiation-induced brain injury, brain atrophy, and pseudoprogression. With the advancement of accelerator neutron source technology, improved targeting of boron carriers, and enhanced precision of treatment planning, the BNCT is expected to break through existing limitation and become an important supplement to the comprehensive treatment of GBM. However, its long-term efficacy and safety still need to be further verified through large-scale, multicenter, randomized controlled studies to facilitate its standardized application in neuro-oncology treatment.

### Clinical trials of head and neck carcinoma

Based on the TNM (tumor, node, metastasis) staging system, over 60% of patients with head and neck squamous cell carcinoma (HNSCC) are diagnosed at stage III or IV. Among those treated with surgery and/or chemoradiotherapy, 15% to 40% experience local recurrence, and the risk of distant metastasis remains high [92]. It is reported that the 5-year OS rate for patients with locally recurrent HNSCC is approximately 50% [93]. Treatment strategies for locally recurrent tumors primarily depend on tumor location. For patients who are not candidates for surgery, concurrent chemoradiotherapy is the preferred approach [93]. However, due to the limited radiation tolerance of normal tissues, repeated irradiation is constrained, making it challenging to perform multiple courses of radiotherapy in patients with recurrence.

**Fig. 4.**
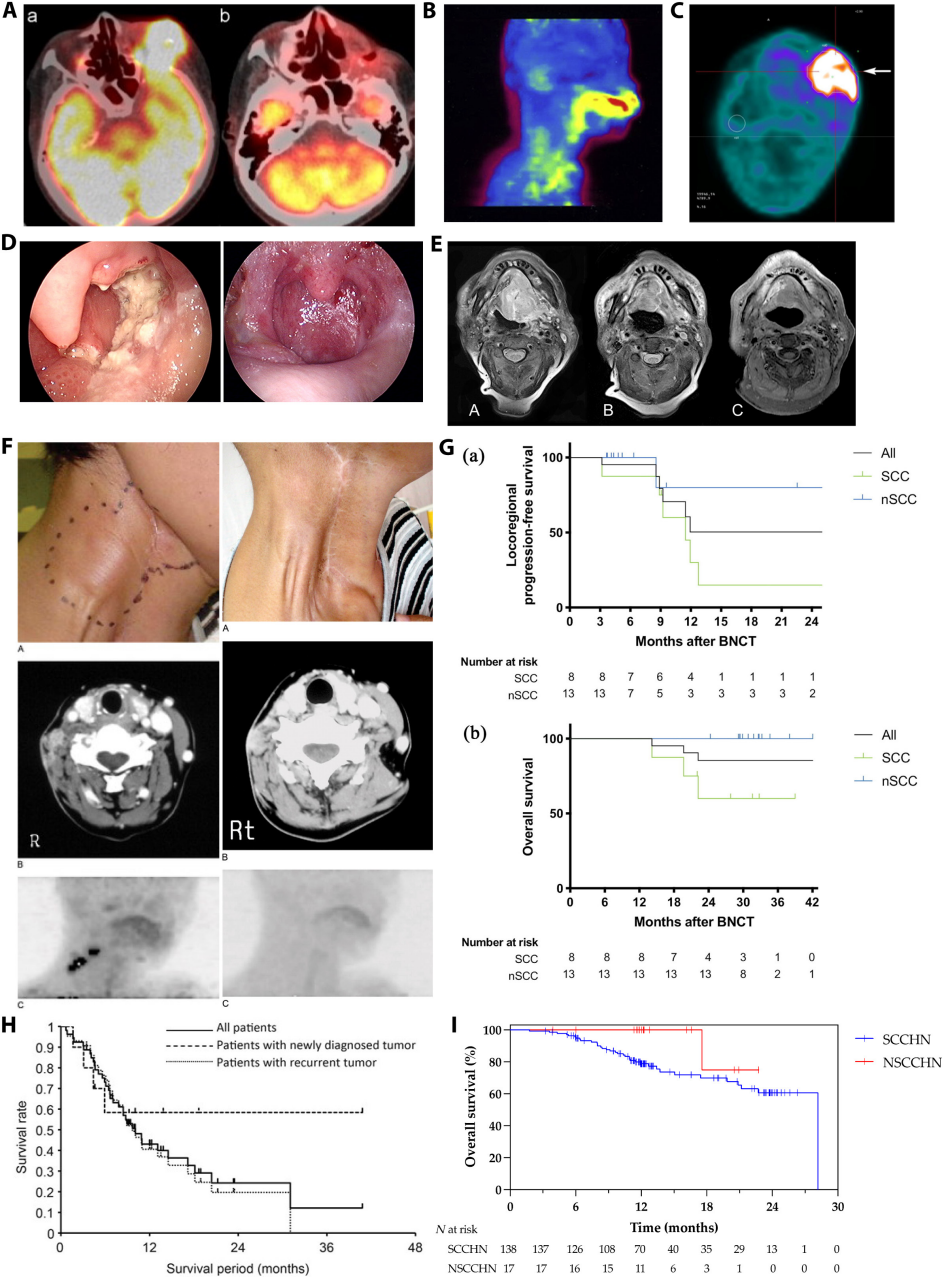
(A) The pre-BNCT FDG-PET study showing FDG tracer accumulation in the left orbital region (arrow) and the frontal lobe of the brain (a); no FDG-PET accumulation was observed in the left orbital region 6 months after BNCT (b) [95]. (B) ^18^F-BPA-PET imaging reveals BPA accumulation in the tumor region [104]. (C) ^18^F-BPA-PET imaging shows higher uptake of BPA in tumor tissue [112]. (D) Gross appearance of recurrent tongue cancer in the left oropharynx and hypopharynx before BNCT (left side); complete tumor remission without recurrence 10 months after BNCT (right side) [100]. (E) MR images of a patient with local recurrence of tongue cancer during BNCT treatment [108]: before BNCT (A), tumor volume reduction 3 months after BNCT (B), and CR 2 years after BNCT (C). (F) Gross appearance, MRI, and ^18^F-BPA PET imaging of a patient with recurrent submandibular gland carcinoma before and after BNCT [101]: left side (A to C) shows pre-BNCT images; right side (A to C) shows images 12 months after BNCT, with complete tumor disappearance and no BPA accumulation in the lesion. (G) Kaplan–Meier curves for local-regional control and OS in patients with recurrent squamous cell carcinoma (R-SCC) and recurrent/locally advanced non-squamous cell carcinoma (R/LA-nSCC) [116]: (a) local–regional progression-free survival and (b) OS. (H) Retrospective analysis of OS after BNCT in 62 patients with head and neck tumors, including all patients (solid line), newly diagnosed tumor patients (dashed line), and recurrent tumor patients (dotted line) [103]. (I) Survival curves for 144 patients with squamous cell carcinoma of the head and neck (SCCHN) and 17 patients with non-squamous cell carcinoma of the head and neck (NSCCHN); the 1- and 2-year OS rates for recurrent SCCHN patients were 78.8% and 60.7%, respectively [125].

The BNCT enables the selective concentration of radiation doses within tumor cells that have absorbed boron-containing agents, making it a promising alternative treatment for locally recurrent head and neck carcinoma (Table 2). Kato was the first to apply BNCT in the treatment of 6 cases of recurrent malignant head and neck carcinoma using BSH and BPA at the KURR. The tumor volume reduction rate in these patients ranged from 46% to 100%, accompanied by improved quality of life and minimal side effects. Among them, the first patient, who had recurrent parotid gland carcinoma, experienced a 63% reduction in tumor volume after the initial BNCT. This patient underwent 3 BNCT sessions in total, achieving effective tumor control with locoregional disease control maintained for up to 7 years [94]. Subsequently, between December 2001 and December 2007, Kato et al. [95] treated 26 patients with recurrent malignant head and neck carcinoma at the KURR and the JRR-4 using BSH and BPA, followed by neutron irradiation therapy. Pretreatment ^18^F-BPA-PET scans confirmed BPA accumulation in the tumor area (Fig. 4A). Among all patients, 12 achieved CR, 10 had PR, 3 experienced PD, and 1 was not evaluated, resulting in an overall response rate of 85%. Nine patients (35%) were disease-free survivors, and the average survival time after BNCT was 13.6 months. To evaluate whether the intra-arterial administration system is superior to the intravenous administration system, Suzuki et al. [96] performed BNCT treatment on 10 evaluable patients with recurrent head and neck cancer. Among the 6 patients in the intravenous group, 2 exhibited PR, 3 had SD, and 1 experienced PD. In the intra-arterial group of 4 patients, 1 achieved CR and 3 had PR. Between February 2004 and December 2007, Wittig et al. conducted a phase I clinical trial to explore the distribution of BPA and BSH in patients with HNSCC, enrolling a total of 6 patients. Three patients were administered BPA before surgery, and another 3 were given BSH. The concentration of ^10^B in different tissues was measured using prompt gamma-ray spectroscopy [97,98]. After the infusion of BPA, the ^10^B concentration ratio of tumor to blood was 4.0 ± 1.7, whereas after BSH infusion, the ratio was 1.2 ± 0.4. These findings indicate that both BPA and BSH can deliver ^10^B to HNSCC [99]. Kankaanranta et al. [100] evaluated the safety and efficacy of BNCT for head and neck cancer that recurred after conventional radiotherapy by conducting a prospective, single-center phase I/II study. Between 2003 December 1 and 2005 December 31, a total of 12 patients with locally recurrent head and neck carcinoma were treated. After intravenous infusion of BPA, neutron beam irradiation was delivered at the Finnish FiR 1, a 250-kW Triga Mark II nuclear research reactor. Seven (58%) patients achieved CR, 3 (25%) patients achieved PR, and 2 (17%) patients had SD for durations of 5.5 and 7.6 months, respectively. The median duration of CR was 14.0 months, and the median duration of PR was 6.8 months. The median time to disease progression was 9.8 months, and the median OS time was 13.5 months. At the time of analysis, 4 patients (33%) were still alive and recurrence-free (Fig. 4D). Aihara et al. [101] reported the clinical outcomes of BNCT for recurrent submandibular gland carcinoma. On the second day after treatment, patients exhibited erythema and edema at the irradiated site, which gradually disappeared within 10 d, leading to tumor volume reduction. Approximately 2 months later, the tumor had almost completely regressed. Imaging studies confirmed no recurrence or tumor residue after 12 months (Fig. 4F). No neurological toxicities were observed throughout the treatment period. Furthermore, between October 2003 and September 2007, Aihara et al. [102] treated 20 patients with advanced or recurrent head and neck carcinoma, achieving CR in 11 patients, PR in 7 patients, and SD in 2 patients, resulting in an ORR of 90%. The 1- and 2-year OS rates for all patients were 47.1% and 31.4%, respectively, while the 1- and 2-year disease-free survival rates were 22.0% and 0%, respectively. The 1- and 2-year locoregional PFS (LPFS) rates were 55.9% and 22.4%, with a median OS of 18.8 months. Distant metastasis was identified as the primary cause of death. Suzuki et al. [103] also retrospectively analyzed the efficacy of BNCT in 62 patients with advanced or recurrent head and neck carcinoma treated between December 2001 and September 2007. The survival data of 53 patients were available, with a median follow-up time of 18.7 months. Among the 57 patients analyzed for treatment response, 16 (28%) achieved CR and 17 (30%) achieved PR. The ORR within 6 months after BNCT was 58%. The MST was 10.1 months, with 1- and 2-year OS rates at 43.1% and 24.2%, respectively (Fig. 4H). Kimura et al. [104] reported the first successful case of BNCT treatment for a patient with papillary cystadenocarcinoma of the upper lip, who underwent 2 BNCT irradiation sessions spaced 4 weeks apart. Pretreatment ^18^F-BPA-PET scans revealed BPA accumulation in the tumor area (Fig. 4B). After the first BNCT session, the tumor volume shrank, and after the second session, the patient experienced pain relief. Five months after the second BNCT session, MRI demonstrated an 86% reduction in tumor size. At the time of reporting, the tumor was nearly invisible to the naked eye, the patient’s pain had disappeared, and he was able to wear a full denture, resulting in an improved quality of life. Additionally, Kimura et al. [105] treated 6 patients with recurrent oral cancer that metastasized to the cervical lymph nodes at the KURR. Five patients experienced immediate relief from spontaneous pain, and improvements in eating difficulties were observed. Three of the patients survived for 23 to 29 months. Haginomori et al. [106] treated a patient with extensively metastatic squamous cell carcinoma of the temporal bone in January 2007, which had recurred following surgery, conventional radiotherapy, and chemotherapy. After intravenous infusion of BPA, the patient received 2 BNCT sessions at the Japanese JRR-4, 1 month apart, targeting both superficial and deep tumor sites. Pain relief was achieved after the first BNCT session, and imaging studies confirmed tumor reduction. One month later, the patient received a second BNCT session and experienced reduced pain, and a mass of necrotic tumor was aspirated through a venous fistula. A computed tomography (CT) scan conducted 6 months after the first BNCT session showed a marked reduction in the middle ear tumor, while a follow-up ^18^F-BPA-PET scan revealed no evidence of tumor recurrence. Ariyoshi et al. [107] reported the clinical outcomes of BNCT in 4 patients with recurrent oral cancer, with 3 cases achieving PR and 1 case PD. One patient experienced a substantial reduction in neck mass and alleviated pain, resulting in a marked improvement in quality of life. Koivunoro et al. [108] conducted a retrospective analysis of neutron irradiation therapy at the Finnish FiR 1 BNCT facility from February 2003 to January 2012. A total of 79 patients with inoperable, locally recurrent HNSCC were treated. Among these, 25 cases (36%) achieved CR (Fig. 4E), 22 cases (32%) achieved PR, 17 cases (25%) had SD, with a median duration of 4.2 months, and 5 cases (7%) experienced PD. The median follow-up time after BNCT was 7.8 years. The 2-year LPFS rate was 38%, and the OS rate was 21%. Additionally, Koivunoro et al. [109] investigated the correlation between T/N ratios and treatment outcomes in 31 patients with locally recurrent head and neck carcinoma treated with neutron irradiation at the Finnish FiR 1 BNCT facility from February 2003 to 2012. The median OS for all patients was 22.8 months, with a 2-year OS of 42%. The median OS for patients with T/N >2.7 was 12.3 months, while the median OS for patients with lower T/N ratios was 27.7 months. The relationship between high T/N ratios on ^18^F-BPA-PET and poor survival rates after BNCT requires further experimental validation. In terms of combined therapy, Kankaanranta et al. [110] reported the therapeutic effects of BNCT combined with chemotherapy and image guided-intensity modulated radiotherapy (IG-IMRT). The patient’s tumor was near both optic nerves, hence inoperable and unsuitable for conventional radiotherapy. Two weeks after BNCT, a CT scan showed a reduction in tumor volume. One month later, the patient received IMRT combined with chemotherapy, achieving radiological CR 3 months post-BNCT. At 10 months after BNCT, endoscopic examination and biopsy showed no evidence of disease, with normal vision maintained.

**Table 2.** Clinical trials of head and neck tumors

Author	Time	Country/region	Boron drug	Neutron source	Neutron type	Tumor type	Outcomes	Number of cases
Kato et al. [95]	2001–2007	Japan	BPA, BSH	KURR, JRR-4	Epithermal	Recurrent HNSCC/SGC/sarcoma	CR: 12 cases; PR: 10 cases; PD: 3 cases; not evaluated: 1 case; ORR: 85%; average survival time: 13.6 months	19/4/3
Suzuki et al. [103]	2001–2007	Japan	BPA, BSH	KURR	Epithermal	Recurrent HNSCC	CR rate: 28%; PR rate: 30%; MST: 10.1 months; 1-year OS rate: 43.1%; 2-year OS rate: 24.2%	62
Aihara et al. [101]	2003	Japan	BPA	KURR	Epithermal	Recurrent submandibular gland carcinoma	Tumor had almost completely regressed	1
Kankaanranta et al. [100]	2003–2005	Finland	BPA	FiR 1	Epithermal	Locally recurrent HNC	CR rate: 58%; PR rate: 25%; PD rate: 17%	12
Aihara et al. [102]	2003–2007	Japan	BPA	JRR-4	Epithermal	Advanced or recurrent head and neck cancer	CR: 11 cases; PR:7 cases; PD: 2 cases MST: 18.8 months	20
Koivunoro et al. [108]	2003–2012	Finland	BPA	FiR 1	Epithermal	Locally recurrent HNC	CR rate: 36%; PR rate: 32%; SD rate: 25%; 2-year LPFS: 38%; OS rate: 21%	79
Suzuki et al. [96]	2004	Japan	BPA	KURR	Epithermal	Recurrent HNSCC	Intravenous group: 2 cases of PR, 3 cases of no change, and 1 case of PD; intra-arterial group: 1 case of CR and 3 cases of PR	10
Kimura et al. [104]	2005	Japan	BPA	KURR	Epithermal	Upper lip papillary cystadenocarcinoma	Tumor remission	1
Kimura et al. [105]	2007	Japan	BPA, BSH	KURR	Epithermal	Recurrent oral cancer	Three patients survived for 23–29 months	6
Haginomori et al. [106]	2007	Japan	BPA	JRR-4	Epithermal	Recurrent temporal bone squamous cell carcinoma	Tumor shrank obviously after 6 months of BNCT	1
Ariyoshi et al. [107]	2007	Japan	BPA	KURR	Epithermal	Recurrent oral cancer	PR: 3 cases; PD: 1 case	4
Kankaanranta et al. [110]	2010	Finland	BPA	FiR 1	Epithermal	HNC	Radiological complete response at 3 months post-BNCT.	1
Wang et al. [111]	2010–2015	Taiwan province of China	BPA	THOR	Epithermal	Locally recurrent HNC	CR: 6 cases; PR: 6 cases; 2-year OS rate: 47%; 2-year local control rate: 28%	17
Wang et al. [112,115]	2014–2021	Taiwan province of China	BPA	THOR	Epithermal	Locally recurrent HNC	CR: 3 cases; PR: 3 cases; SD: 3 cases; 1-year OS rate: 56%	14
Hirose et al. [116–118]	2016–2018	Japan	BPA	C-BENS (AB-BNCT)	Epithermal	Recurrent HNSCC	Overall ORR: 71%; CR/PR rates of 50%/25% for R-SCC and 8%/62% for R/LA-nSCC.	21
Hirose and Sato [119]	2020–2021	Japan	Borofalan	NeuCure BNCT System (AB-BNCT)	Epithermal	Recurrent HNSCC	CR rate: 51%; overall response rate: 74%; 1-year disease-free survival rates: 34.6%; 2-year disease-free survival rates: 26.6%	47
Takeno et al. [120,121]	2020–2022	Japan	Borofalan	NeuCure BNCT System	Epithermal	HNC	CR: 32 cases PR: 26 cases; SD: 10 cases; PD: 2 cases ORR: 80.5%; 1-year local control rate: 57.1%; progression-free survival rate: 42.2%	69
Sato and Hirose [122]	2020–2022	Japan	Borofalan	NeuCure BNCT System	Epithermal	HPC/LCA	CR: 27 cases; PR: 4 cases; ORR: 86.1%; MST: 15.5 months; 1-year OS rates: 87.6%; 2-year OS rates: 79.8%	25/11
Sato et al. [125]	2020	Japan	Borofalan	NeuCure BNCT System	Epithermal	SCCHN/NSCCHN/glioblastoma	SCCHN: ORR (72.3%), CR rate (t 46.0%), PR rate (26.3%) NSCCHN: CR rate (47.1%), PR rate (17.7%), ORR rate (64.7%)	144/17/1

FiR 1, Reactor in Espoo, Finland; HNSCC, head and neck squamous cell carcinoma; SGC, salivary gland carcinoma; HNC, head and neck cancer; HPC, hypopharyngeal cancer; LCA, laryngeal cancer; R-SCC, recurrent squamous cell carcinoma; R/LA-nSCC, recurrent/locally advanced non-squamous cell carcinoma; PR, partial response; LPFS, locoregional progression-free survival rate; MST, median survival times; OS, overall survival; SD, stable disease

Wang et al. [111] from Taipei Veterans General Hospital initiated the use of BNCT for patients with head and neck cancer in August 2010. Between August 2010 and June 2015, a total of 17 patients with locally recurrent head and neck carcinoma were treated. Among them, 15 received 2 BNCT sessions, spaced 30 d apart, after intravenous infusion of BPA and neutron irradiation treatment at THOR. With a median follow-up time of 19.7 months, 6 patients achieved CR and another 6 achieved PR. Of the 14 patients with tumor volumes less than 20 cm^3^, 8 (57%) reached CR; among the 6 participants who received a total D80 > 40 Gy-Eq, 4 (67%) achieved CR. The 2-year OS rate for all patients was 47%, the 2-year local control rate was 28%, 2 patients survived for more than 4 years, and both were disease-free at 50 and 77 months post-BNCT. In investigating the therapeutic efficacy of combining IG-IMRT with BNCT, Wang’s team [112] recruited 9 patients with locally recurrent head and neck carcinoma in 2014. Of these, 7 received combined treatment IMRT 28 d after BNCT, while 2 patients received BNCT alone. The pre-BNCT ^18^F-BPA-PET scans showed high BPA uptake in tumor cells (Fig. 4C). The median follow-up time was 11.7 months, with 3 patients achieving CR and 3 patients achieving PR after combined treatment, and the remaining 3 patients had SD. The 1-year OS rate for all patients was 56%, with one patient being disease-free 25.9 months after combined treatment, and local recurrence remaining the main cause of treatment failure. Lee et al. [113] examined the dose distribution of the GTV in 9 patients with locally recurrent head and neck carcinoma. The results showed that BNCT combined with compensatory fractionated IMRT yielded higher uniformity and consistency in dose distribution compared to BNCT alone, particularly in tumors larger than 100 cm^3^, suggesting that combined therapy may improve local tumor control. Furthermore, Chang et al. [114] from Taiwan province of China reported the therapeutic outcomes of combining BNCT with IG-IMRT in 5 patients with recurrent head and neck carcinoma. Among them, 2 patients with oral cancer experienced disease progression, 2 exhibited tumor volume reduction and achieved CR, and 1 patient exhibited SD with no change in tumor volume. A retrospective analysis conducted at Taipei Veterans General Hospital examined the therapeutic effects of BNCT in patients with recurrent head and neck carcinoma treated between July 2014 and September 2021. The study included 14 cases, 12 of whom underwent combined BNCT and IG-IMRT treatment. The median follow-up period for all patients was 11.8 months. PET-CT scans 3 months after IG-IMRT showed that 5 patients achieved CR and 4 achieved PR. The median time to local tumor progression was 8.3 months. The 1-year LPFS rate for all patients was 21% [95% confidence interval (CI): 5% to 45%], and the 1-year OS rate was 56% (95% CI: 3% to 77%). The 1-year LPFS for patients undergoing combined BNCT and IMRT treatment was 25% (95% CI: 6% to 50%) [115].

Hirose was the first to apply AB-BNCT to treat patients with recurrent squamous cell carcinoma (R-SCC) and recurrent/locally advanced non-squamous cell carcinoma (R/LA-nSCC) of the head and neck. Between June 2016 and February 2018, a total of 21 patients were enrolled, comprising 8 cases of R-SCC and 13 cases of R/LA-nSCC. Borofalan served as the boron delivery agent, and the neutron irradiation treatment was conducted at the Southern Tohoku BNCT Research Center in Japan using the AB-BNCT platform. Among the 21 patients, 20 achieved local tumor control within the first 3 months; the overall ORR for all patients was 71%, with CR/PR rates of 50%/25% for R-SCC and 8%/62% for R/LA-nSCC. The 2-year OS rates were 58% for R-SCC and 100% for R/LA-nSCC. For patients with R-SCC, the median PFS was 11.5 months, and the median follow-up time was 24.2 months (Fig. 4G), and a reduction in tumor volume was observed in all patients within 90 d after BNCT [116–118]. Hirose and Sato [119] also retrospectively analyzed the clinical outcomes of AB-BNCT treatment for recurrent HNSCC between May 2020 and February 2021 in Japan. After treating 47 patients with recurrent HNSCC, the CR rate was 51%, and the ORR was 74%; the 1- and 2-year disease-free survival rates were 34.6% and 26.6%, respectively; the 1- and 2-year OS rates were 86.1% and 66.5%, respectively. Among patients who achieved CR after BNCT, the 2-year OS rate was 78.9%, which was notably higher than the 49.4% observed in patients who did not achieve CR. Takeno et al. [120,121] explored the clinical outcomes of BNCT treatment for head and neck cancer and treated a total of 69 patients from June 2020 to May 2022. After intravenous infusion of borofalan, the AB-BNCT system (NeuCure BNCT System) was used as the neutron source for neutron irradiation treatment. The median observation period was 15 months, with 32 cases of CR, 26 cases of PR, 10 cases of SD, and 2 cases of PD, resulting in an ORR of 80.5%, a 1-year local control rate of 57.1% (95% CI: 44.4% to 68.7%), a PFS rate of 42.2% (95% CI: 30.7% to 54.3%), and an OS rate of 75.4% (95% CI: 63.3% to 84.8%). Patients with earlier TNM staging and no history of chemotherapy had considerably prolonged local control times. Sato and Hirose [122] conducted a retrospective analysis to evaluate the efficacy and safety of BNCT for patients with hypopharyngeal cancer (HPC)/laryngeal cancer (LCA) at the Southern Tohoku BNCT Research Center in Japan. Between June 2020 and August 2022, a total of 25 cases of HPC and 11 cases of LCA were treated. After intravenous infusion of borofalan, neutron irradiation treatment was performed using AB-BNCT as the neutron source. Among the 36 patients, 27 (75.0%) achieved CR, 4 achieved PR, with an ORR of 86.1% at 3 months, and no cases of PD were observed; the 1-year local control rate was 63.1%, the 1-year PFS rate was 53.7%, and the median PFS was 13.6 months; the median OS was 15.5 months, with 1- and 2-year OS rates of 87.6% and 79.8%, respectively; among the 27 patients who achieved CR, 11 relapsed, with a median time to relapse of 6.0 months. The most common treatment-related adverse events in the acute phase were pharyngitis (100%), alopecia (81%), oral mucositis (78%), dysgeusia (64%), and sialadenitis (50%). No grade 4 or 5 adverse events were observed during the acute phase, except for hyperamylasemia. Additionally, no grade 3 or higher adverse events were observed in the late phase. Higashino et al. [123] conducted a retrospective analysis to evaluate the therapeutic effects of BNCT treatment in 15 patients with residual or recurrent LCA after radical radiotherapy. The ORR 3 months after BNCT was 93.3%, including a CR rate of 73.3%, with 11 cases achieving CR, 3 cases achieving PR, and 1 case showing SD. Antitumor activity was observed as early as the second day after treatment, and the tumor disappeared after 3 months. The most common adverse event related to BNCT was laryngeal edema, which was recoverable within 1 week. These findings suggest that BNCT exhibits promising short-term antitumor effects in recurrent LCA; however, its long-term efficacy requires further investigation. Higashino et al. [124] reported a case of recurrent LCA successfully treated with salvage BNCT. The patient had previously received intensity-modulated radiotherapy and concurrent cetuximab treatment but experienced recurrence in the left supraclavicular lymph node 2 years later, with tumor invasion into adjacent major blood vessels. After the BNCT, the maximum tumor diameter of the tumor decreased to approximately 70% and 50% of the original diameter at the 8th and 20th weeks, respectively. At 24 weeks post-BNCT, the patient underwent salvage surgery. Histopathological examination of the resected tissue revealed squamous cell carcinoma in the center of the tumor tissue and severe fibrosis at the tumor margin. However, the tissue outside the irradiated area exhibited almost no fibrosis. No tumor recurrence was observed during the 2-year follow-up. These findings indicate that BNCT can effectively reduce tumor burden while sparing surrounding normal tissue, providing a promising therapeutic option for patient ineligible for conventional surgery.

Sato et al. [125] conducted a prospective, multicenter observational study to monitor the therapeutic effects of AB-BNCT in combination with borofalan after its market release. A total of 162 patients were enrolled, including 144 patients with squamous cell carcinoma of the head and neck (SCCHN), 17 patients with non-squamous cell carcinoma of the head and neck (NSCCHN), and 1 patient with GBM. Reported acute treatment-related adverse events included hyperamylasemia (84.0%), stomatitis (51.2%), sialadenitis (50.6%), and alopecia (49.4%). The common late treatment-related adverse events were dysphagia (4.5%), thirst (2.6%), and dermatitis (1.9%). For the 137 evaluable SCCHN patients, the ORR was 72.3%, with CR at 46.0% and PR at 26.3%. For the 17 patients with NSCCHN, the CR was 47.1%, PR was 17.7%, and ORR was 64.7%. The 1- and 2-year OS rates for recurrent SCCHN patients were 78.8% and 60.7%, respectively, while the 1-year OS rate for NSCCHN patients was 100% (Fig. 4I). Consistent results from multiple small-sample clinical studies indicate that BNCT holds potential therapeutic efficacy for recurrent head and neck tumors, with the ability to extend the survival period and improve the quality of life in some patients. In June 2020, Japan became the first country to incorporate BNCT into its national health insurance coverage for locally advanced or recurrent head and neck tumors. The clinical advancement of accelerator-based neutron sources and boron carriers like borofalan is paving the way for BNCT to transition from reactor-based systems to routine clinical settings.

A clinical trial conducted at Xiamen Hongai Hospital using the accelerator-based “Jifeng Knife” AB-BNCT system further demonstrated a highly favorable radiation safety profile. The study found that the main radionuclides produced by neutron activation (^24^Na, ^38^Cl, and ^49^Ca) in patients cleared rapidly and completely. Within just 20 min post-treatment, the ambient dose equivalent rate at 1 m from the irradiation site was measured to be below 2.5 μSv/h. These findings strongly confirm that AB-BNCT, as delivered by this system, poses a very low radiological risk, ensuring a controllable and safe environment for medical staff and family members [126]. This shift provides a new treatment option for patients who have previously failed radiotherapy, are unsuitable for surgery, or have limited organ function, offering a form of precision radiotherapy that is low in toxicity, highly efficient, and repeatable.

### Clinical trials of meningioma

Meningioma is the most common primary intracranial tumor in adults. According to the 2021 WHO classification, it can be divided into benign meningioma (WHO grade 1), atypical meningioma (WHO grade 2), and anaplastic meningioma (WHO grade 3), with grades 2 and 3 considered invasive [127]. Most meningiomas are benign and exhibit a slow growth rate; therefore, primary management strategies include routine monitoring and symptomatic treatment. Surgical resection is the preferred treatment upon disease progression, while radiotherapy is typically reserved for cases where surgery is not feasible [128]. However, for recurrent meningiomas, since the tumor can recur at the original site and the cumulative radiation dose to the tissue cells is limited, repeated radiotherapy cannot be performed. The BNCT can concentrate the radiation dose within tumor cells that take up boron-containing drugs, making it a promising therapeutic option for recurrent meningiomas (Table 3).

**Table 3.** Clinical trials of meningioma

Author	Time	Country/region	Boron drug	Neutron source	Neutron type	Tumor type	Outcomes	Number of cases
Tamura et al. [129]	2005	Japan	BPA, BSH	KURR	Epithermal	Recurrent meningioma	Tumor volume shrank	1
Miyatake et al. [132]	2002–2007	Japan	BPA, BSH	KURR	Epithermal	Malignant meningioma/glioblastoma	Pseudoprogression: 3 patients	13/52
Stenstam et al. [130]	2003–2007	Sweden	BPA	Studsvik BNCT Facility	Epithermal	Recurrent intracranial meningioma	Case 1: recurrence outside irradiated area; case 2: recurrence at 6 months post-treatment	2
Miyatake et al. [131]	2005–2006	Japan	BPA	KURR	Epithermal	Meningioma	CR: 2 patients	6
Kawabata et al. [133]	2005–2011	Japan	BPA	KURR	Epithermal	Recurrent meningioma	MST: 14.1 months	20
Takeuchi et al. [135]	2005–2014	Japan	BPA, BSH	KURR	Epithermal	High-grade meningioma	MST: 24.6 months	31
Takai et al. [136]	2005–2019	Japan	BPA, BSH	KURR	Epithermal	Recurrent and refractory high-grade meningioma	Median OS: 29.6 months median PFS: 13.7 months	44
Aiyama et al. [134]	2010–2011	Japan	BPA	JRR-4	Epithermal	Recurrent glioblastoma/anaplastic meningioma	Tumor volume reduces	1/1
Kawaji et al. [137]	2014	Japan	BPA	KURR	Epithermal	Anaplastic meningioma	Liver metastases after 2 months	1
Miyatake et al. [138,139,198,199]	2019–2022	Japan	BPA	AB-BNCT	Epithermal	Recurrent high-grade meningioma	PD: 7 patients median PFS: 90 weeks 2-year OS rates: 91.7%	12
T.Y. Huang et al. [140]	2020–2022	Taiwan province of China	BPA	THOR	Epithermal	Recurrent meningioma	Tumor regression	2
Lan et al. [141]	2020–2023	Taiwan province of China	BPA	THOR	Epithermal	Recurrent meningioma	SD: 2 patients PR: 1 patient; CR: 1 patient; mean PFS: 14.7 months	4

MST, median survival time; OS, overall survival; PFS, progression-free survival

**Fig. 5.**
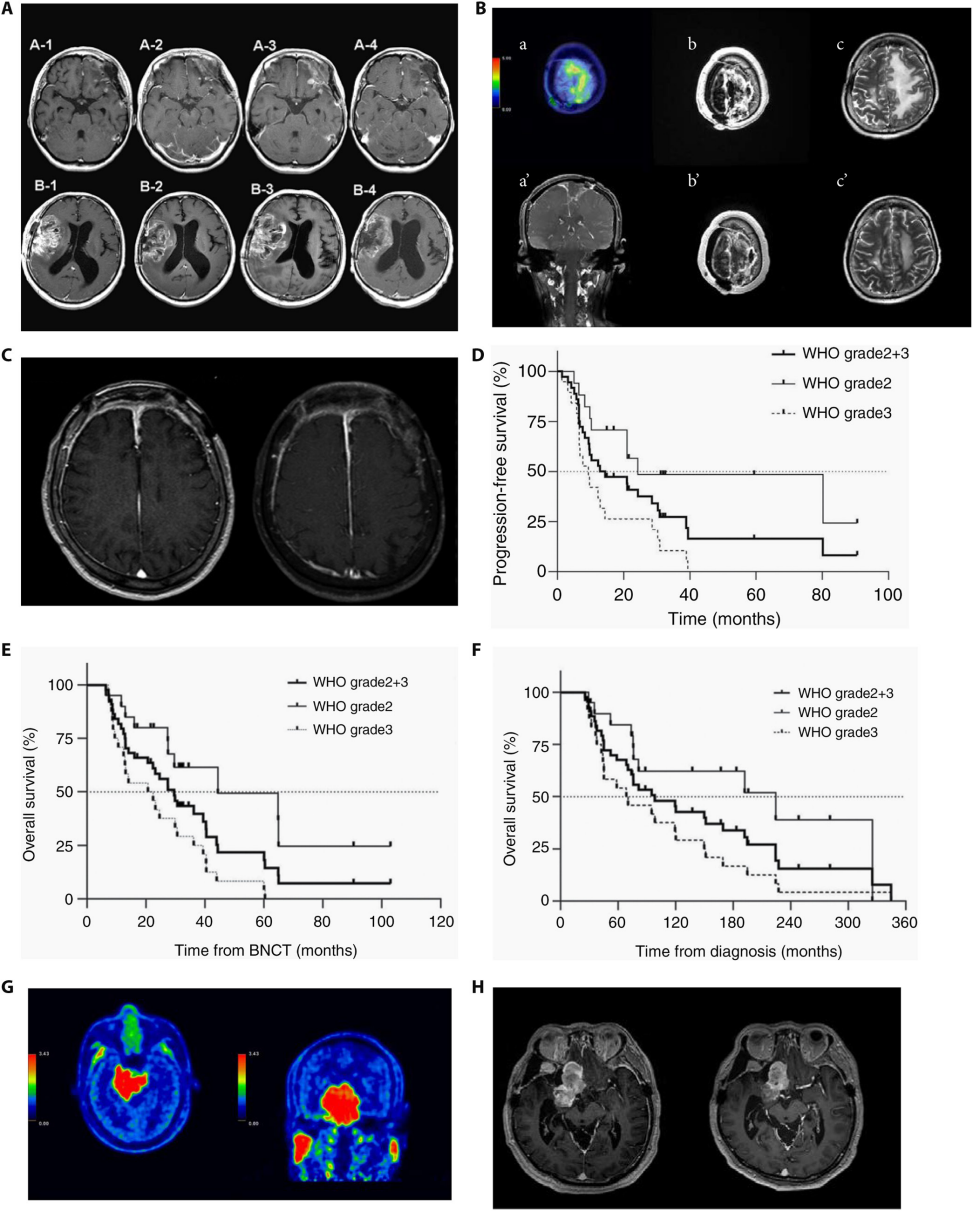
(A) Gd-enhanced MR images of 2 cases in recurrent malignant meningiomas treated with BNCT show a decrease in tumor volume [132]. Case 50: before BNCT (A-1), 48 h after BNCT (A-2), 1 month after BNCT (A-3), and 2 months after BNCT (A-4). Case 56: before BNCT (B-1), 1.5 months after BNCT (B-2), 2.5 months after BNCT (B-3), and 6 months after BNCT (B-4). (B) Follow-up imaging of a patient with recurrent atypical meningioma (upper left frontal region) [141]. Before BNCT, ^18^F-BPA-PET image with a T/N ratio of approximately 2.64 (a); axial MRI contrast scan (b); axial MRI T2-weighted image (c). Two months after BNCT, coronal MRI contrast scan (a′); axial MRI contrast scan showing decreased signal (b′); axial MRI T2-weighted image showing markedly reduced perilesional edema (c′). (C) MRI contrast-enhanced images of malignant meningiomas [134]. Comparison of pre-BNCT (left) and 3 months post-BNCT (right) reveals a reduction in tumor size and decreased uptake. (D) Kaplan–Meier analysis of progression-free survival after BNCT [136]. (E and F) Kaplan–Meier curves after BNCT and following the diagnosis of high-grade meningioma [136]. (G) The pre-BNCT ^18^F-BPA PET scan showed a T/N uptake ratio of 2.89 and physiological uptake in the parotid glands [141]. (H) Contrast-enhanced MRI before BNCT (left) and at the 2-month follow-up (right) shows stable tumor size and partial contrast enhancement regression was observed [141].

Tamura et al. [129] reported the first case of a patient with recurrent meningioma treated with BNCT. BPA and BSH were used as boron carriers, and the patient received 2 sessions of BNCT. Pretreatment ^18^F-BPA-PET imaging revealed substantial BPA uptake in the right frontal lobe lesion. One week after the initial BNCT session, the patient was able to walk without a wheelchair. By the seventh week, neurological deficits had resolved; 20 weeks later, the tumor recurred in the left frontal lobe and extended outside the skull; 22 weeks later, the patient underwent the second BNCT targeting the left frontal lobe lesion, after which the lesion shrank. After 2 sessions of BNCT, the tumor volume markedly decreased from 65.6 cm^3^ at the time of the first BNCT to 31.8 cm^3^ at 26 weeks later. These results suggest that BNCT may be a potential new treatment for patients with recurrent meningiomas. Stenstam et al. [130] explored the efficacy of BNCT by treating 2 patients with recurrent intracranial meningiomas at the Studsvik reactor in Sweden. The first patient had good quality of life for 22 months after treatment. Tumor recurrence was observed outside the irradiated region at 30 months, while no recurrence occurred at the original site. The patient eventually died 32 months after BNCT. The second patient had a recurrence 6 months after treatment, which was successfully removed by surgery, and had good quality of life within 26 months after BNCT. Miyatake et al. [131] attempted to control meningioma-associated malignancies using BNCT. Among 6 treated patients, 5 exhibited favorable BPA uptake with a T/N ratio greater than 2.7. Of the 3 patients diagnosed with anaplastic meningioma, 2 achieved CR. Imaging of the 6 evaluable patients showed tumor shrinkage, and all but one patient experienced symptom alleviation. Additionally, Miyatake et al. [132] conducted a retrospective analysis of BNCT treatment outcomes in 13 patients with malignant meningioma. These patients exhibited high uptake of BPA in their tumors, with T/N ratios all exceeding 2.5. Pseudoprogression was observed in 3 patients within 3 months after BNCT (Fig. 5A). Kawabata et al. [133] treated 20 patients with recurrent meningioma via BNCT from June 2005 to September 2011. Eighteen patients had good BPA uptake, with a T/N ratio greater than 2.7. Post-treatment, the average tumor volume reduction was 64.5% within 2 months. The median OS after BNCT and diagnosis was 14.1 and 45.7 months, respectively, with 6 patients remaining alive at the time of reporting. Moreover, symptoms such as hemiplegia and facial pain showed clinical improvement. Aiyama et al. [134] reported the outcomes of BNCT in 2 patients with brain tumors. One of these patients was diagnosed with atypical meningioma. Post-treatment follow-up MRI and ^11^C-Met-PET scans revealed a reduction in tumor volume and uptake, with no severe adverse events observed (Fig. 5C). Takeuchi et al. [135] retrospectively analyzed BNCT outcomes in 31 patients with high-grade skull-base meningioma treated at KURR in Japan. Patient exhibited good BPA uptake, with boron concentrations approximately 3.8 times higher than those in normal brain tissue. During follow-up, tumor volume shrank, and several cases experienced pseudoprogression. The median OS for patients with skull-base meningioma after BNCT and after diagnosis of high-grade meningioma was 24.6 and 67.5 months, respectively. Takai et al. [136] conducted a retrospective analysis of 44 patients with recurrent and refractory high-grade meningioma who underwent reactor-based BNCT. Among the 44 patients, 20 (45.5%) had WHO grade 2 tumor and 24 (54.5%) had WHO grade 3 tumor. Post-BNCT, 35 patients exhibited tumor shrinkage during the follow-up period. The median OS was 29.6 months, and the median OS from initial diagnosis was 98.4 months. The median follow-up time after BNCT was 26 months, with a local recurrence rate of only 22.2%. The median OS after BNCT for WHO grade 2 and grade 3 patients was 44.4 and 21.55 months, respectively, with grade 2 patients having markedly longer median OS than grade 3 patients (Fig. 5E and F). The median PFS for 36 patients after BNCT was 13.7 months. The median PFS for grade 2 and grade 3 patients after BNCT was 24.3 and 9.4 months, respectively (Fig. 5D), with grade 2 patients having substantially longer median OS from diagnosis than grade 3 patients. Kawaji was the first to describe the pathological changes in anaplastic meningioma following BNCT treatment. One month following BNCT, enhanced MRI scans showed a slight reduction in both lesion enhancement and surrounding edema. However, 2 months after treatment, the patient developed multiple liver metastases, with dissemination to the pleura and peritoneum. The patient died 3 months after treatment. Histopathological examination of brain tissue demonstrated that the proliferative activity of meningioma treated with BNCT was markedly lower than that observed in untreated metastatic liver tumors and meningioma specimens collected during the second surgery. These findings support the antitumor efficacy of BNCT [137]. In 2019, a phase II clinical trial employing the AB-BNCT system for refractory recurrent high-grade meningioma was initiated in Japan. By 2021, a total of 18 participants were enrolled, with 12 receiving BNCT. One patient withdrew consent and was excluded from the study. The remaining 6 participants were allocated to a control group receiving best supportive care. Follow-up continued until 2023 April 20. In the control group, all 6 patients exhibited PD. Among the 11 evaluable patients in the BNCT group, 7 experienced PD. The median PFS was 8 weeks in the control group and 90 weeks in the BNCT group (*P* = 0.0004). The 2-year OS rates were 91.7% in the BNCT group and 25% in the control group (*P* = 0.01) [138,139].

In Taiwan province of China, Huang et al. [140] reported on the nursing outcomes of 2 cases of salvage BNCT treatment for recurrent meningioma. The T/N ratios of ^18^F-BPA-PET for the 2 patients before BNCT were 2.89 and 2.64. The first patient was diagnosed with a skull-base meningioma. Follow-up MRI revealed PR of the tumor, accompanied by alleviation of dizziness and gradual improvement in gait stability. The second patient was diagnosed with an atypical meningioma. Four months after BNCT, follow-up MRI showed a near CR of the tumor, along with relief of headache and gradual recovery of lower limb muscle strength. From August 2020 to January 2023, Lan et al. [141] enrolled 4 patients with recurrent meningioma to receive BNCT. The T/N ratio of BPA uptake before irradiation was 2.89 (Fig. 5G). After treatment, 2 patients achieved SD, 1 achieved PR, and 1 achieved CR (Fig. 5B and H), with a median PFS of 14.7 months. Clinical trials on the treatment of meningiomas have consistently demonstrated that BNCT is highly effective, particularly in patients with recurrent meningiomas where traditional treatment methods have limited efficacy. The results indicate that BNCT can effectively reduce tumor volume and improve the quality of life for patients. This finding offers a potential therapeutic option for patients with recurrent meningiomas and highlights the considerable potential of BNCT in the future treatment of meningiomas. However, the long-term efficacy and safety of BNCT still need to be further verified through additional studies. Despite this, the current research findings have already provided strong support for the application of BNCT in the treatment of meningiomas.

### Clinical trials for malignant melanoma

Melanoma is a malignant tumor originating from melanocytes, which are cells that produce melanin and are primarily located in the skin, but can also be found in the eyes, gastrointestinal tract, leptomeninges, as well as the mucosa of the oral cavity, genitalia, and nasal sinuses [142]. Cutaneous melanoma is the most common type, and the current first-line treatment is wide surgical excision, with or without lymph node dissection. When local control is not feasible, neoadjuvant chemotherapy, immunotherapy, or advanced radiotherapy modalities such as carbon ion or proton therapy may be employed [142]. However, malignant melanoma is a highly aggressive tumor that frequently metastasizes through the lymphatic system at an early stage. Invasion of adjacent tissues may cause functional, aesthetic, and psychological impairments, thereby reducing patients’ quality of life [143]. The BNCT offers the potential to preserve normal tissue architecture and improve quality of life by achieving effective local control of tumor cells. Table 4 summarizes all clinical trial characteristics in malignant melanoma.

**Table 4.** Clinical trial characteristics in malignant melanoma

Author	Time	Country/region	Boron drug	Neutron source	Neutron type	Tumor type	Outcomes	Number of cases
Mishima et al. [144–146]	1987	Japan	BPA	Musashi reactor	Epithermal	Metastatic melanoma	Lesion regressed	1
Mishima et al. [144]	1988	Japan	BPA	KURR	Epithermal	Primary melanoma	Complete regression	1
Mallesch et al. [147]	1994	Australia	BPA	—	—	Metastatic melanoma/glioblastoma	—	12/6
Hiratsuka et al. [148]	1995	Japan	BPA	Musashi reactor	Epithermal	Primary melanoma	Complete regression	1
Madoc-Jones et al. [149]	1996	USA	BPA	MITR	Epithermal	Malignant melanoma	Response: 2 cases	3
Fukuda et al. [150]	1987–2001	Japan	BPA	KURR	Epithermal	Malignant melanoma	CR: 16 cases; PR: 5 cases	22
González et al. [151]	2003–2004	Argentina	BPA	RA-6 reactor	Epithermal	Recurrent melanoma	21 nodes reached CR; 1 node showed PR	1
Menéndez et al. [152]	2003–2007	Argentina	BPA	RA-6 reactor	Epithermal	Melanoma	ORR: 69.3%	7
Hiratsuka et al. [153]	2003–2014	Japan	BPA	KURR	Epithermal	Primary cutaneous melanoma	CR: 6 cases; PR: 2 cases	8
Hiratsuka et al. [155]	2005–2014	Japan	BPA	KURR	Epithermal	Vulvar melanoma/extramammary Paget’s disease	Regression	1/3
Santa Cruz et al. [154]	2007	Argentina	BPA	RA-6 reactor	Epithermal	Malignant melanoma	CR: 1 case	2
Omori et al. [156]	2012–2014	Japan	BPA	KURR	Epithermal	Recurrent melanoma	Tumor volume reduction at 1 week	1
Yong et al. [157]	2014–2016	China	BPA	IHNI	Epithermal	Malignant melanoma	Regression	1
Igaki et al. [158,159]	2022	Japan	BPA	CICS-1 (AB-BNCT)	Epithermal	Cutaneous malignant melanoma	Achieved PR within 6 months post-treatment	1

IHNI, in-hospital neutron irradiator

Mishima was the first to propose the application of BNCT for melanoma and initiated its first clinical trial in 1987. The patient presented with a subcutaneous metastatic tumor in the left occipital region 3 years after resection of the primary melanoma on the right toe, with possible skull invasion. The patient subsequently underwent neutron irradiation at the Musashi Reactor in Japan. Approximately 2 months after BNCT, the tumor height decreased from 21 mm to 7.5 mm, accompanied by marked regression of clinical symptoms. Symptoms such as diplopia, nausea, and headache resolved early, with no ulceration or crusting observed. Nine months after treatment, the lesion regressed, and clinical and radiological diagnoses showed only fibrosis remaining with no signs of recurrence [144–146]. Additionally, another patient with primary melanoma and right inguinal lymph node metastasis was treated. Two weeks after a neutron capture therapy session, the lesion regressed, and by 9 weeks, complete clinical regression was observed. A residual 3-mm blue spot remained, which was histologically confirmed to result from melanophage accumulation. Eighteen months after neutron capture therapy, the patient remained in good health with no evidence of recurrence [144]. Mallesch et al. [147] conducted a phase I/II clinical trial to evaluate the biodistribution of BPA melanoma patients and found that 12 melanoma patients had good uptake of BPA. The mean tumor-to-blood boron concentration ratio (T/B) was 4.4 ± 3.2. The maximum T/B ratio observed in brain metastases reached 10, satisfying the threshold required for effective intratumoral boron concentration in BNCT. Hiratsuka treated a patient with malignant melanoma on the left foot using neutron irradiation at the Musashi reactor. One month after the BNCT, the melanoma had markedly regressed with mild skin reactions. Ten months after treatment, the melanoma had disappeared, and no substantial side effects were observed during the treatment period [148]. MIT carried out a phase I dose-escalation trial to assess the radiation toxicity threshold, enrolling 3 patients with malignant melanoma of the lower leg. After a follow-up of 5 to 7 months, no radiation-induced normal tissue reactions or other adverse events related to high oral doses of boron compounds or neutron irradiation were observed. At least 2 of the 3 patients demonstrated a clinical response within the irradiated melanoma nodules [149]. Fukuda et al. [150] treated 22 patients with malignant melanoma using BNCT at KURR in Japan from July 1987 to June 2001, achieving 16 CR and 5 PR, with a local ORR (CR + PR) rate of 95%. Skin damage was within tolerable limits in 16 patients, whereas 6 patients experienced skin toxicity exceeding acceptable levels. Of these, 3 cases were curable, and the remaining patients required skin grafts.

**Fig. 6.**
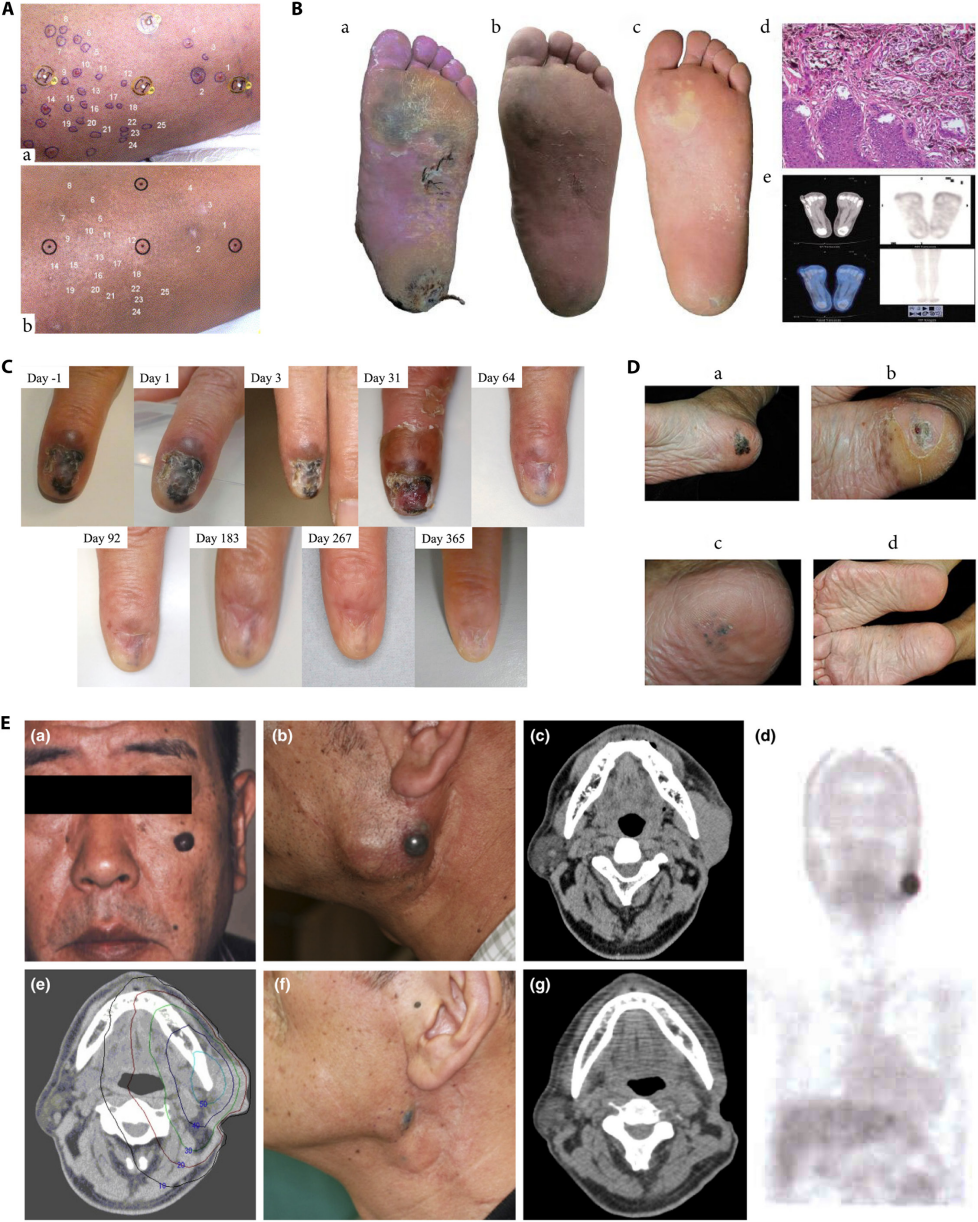
(A) Subcutaneous melanoma nodules before BNCT (a); skin and tumor response at 8 weeks after BNCT with a dose of 15 RBE Gy (b) [151]. (B) Skin and tumor response in a patient with malignant melanoma on the left sole after BNCT [157]: gross examination of the left sole skin lesion at 2 weeks (a), 5 weeks (b), and 24 months (c) after BNCT, showing marked reduction in the lesion size; histopathological analysis of the lesion at 9 months after BNCT, with no nests of pigmented cells or invasion into the deep dermis (d); PET/CT scan at 24 months after BNCT, showing no abnormal FDG uptake (e). (C) Clinical photographs were obtained from a patient with primary melanoma on the left index finger 1 d before administration of BPA and on days 1, 3, 31, 64, 92, 183, 267, and 365 after irradiation. The images demonstrated gradual reduction and disappearance of the tumor lesion, with no severe adverse effects [159]. (D) Therapeutic response in a case of left plantar melanoma [153]: Pre-BNCT gross appearance (a); 1 month post-BNCT, marked tumor shrinkage with dry erosion in the surrounding normal skin (b); 3 months post-BNCT, marked improvement in skin reaction (c); 9 months post-BNCT, complete disappearance of the black spot. The patient experienced no pain while walking. This CR persisted for 5.5 years (d). (E) Therapeutic progression in recurrent left cheek melanoma managed with BNCT [156]: Primary tumor lesion (a); metastatic lesion (b); pre-BNCT CT scan of the metastatic lesion (c); pre-BNCT ^18^F-BPA positron emission tomography image (d); isodose distribution map of tumor cell uptake (biologically equivalent: Gy-eq) (e); marked regression of metastatic lesion at 8 months post-BNCT (f); follow-up CT demonstrating absence of neoplastic recurrence at 8 months post-BNCT (g).

González et al. [151] reported the first clinical outcome of BNCT cutaneous melanoma of the limb in Argentina. The patient, who had recurrence after resection of the primary tumor site and metastatic lymph nodes and observed multiple lymph node progressions following radiotherapy, received neutron irradiation treatment at the Argentine RA-6 reactor. A total of 25 nodes were irradiated. Four weeks after BNCT, 19 nodes achieved CR; 8 weeks later, 21 nodes reached CR, and 1 showed PR (Fig. 6A). A grade 1 Radiation Therapy Oncology Group/European Organisation for Research and Treatment of Toxicity acute skin reaction was detected on the first day of treatment and resolved after 8 weeks. Menéndez et al. [152] also conducted clinical research on BNCT at the Argentine RA-6 reactor. From October 2003 to June 2007, 7 melanoma patients were treated. Among all irradiated nodes, 69.3% showed overall response, 30.7% remained unchanged, and no disease progression was observed within the treated field. Hiratsuka et al. [153] evaluated the long-term clinical outcomes of BNCT in patients with cutaneous melanoma. Between October 2003 and April 2014, 8 patients with primary cutaneous melanoma were treated, with 6 achieving CR and 2 achieving PR. The ORR (CR + PR) was 88%. One of the CR patients maintained the response for 5.5 years (Fig. 6D). Among the 2 patients with PR, one had persistent brown spots for 7.5 years after BNCT, while the other experienced tumor recurrence after 6 years of persistent brown spots. These patients maintained good quality of life, and no grade 2 adverse events were observed during long-term follow-up. Santa Cruz et al. [154] applied BNCT to treat 2 patients with melanoma. The first patient had 2 subcutaneous melanoma nodules on the right thigh, which achieved CR 3 months after BNCT. The second patient had multiple subcutaneous nodules in the lateral malleolar region of the left ankle. After the BNCT, 3 of 10 nodules achieved objective responses (CR and PR), while the remaining nodules remained unchanged. Hiratsuka et al. [155] also treated a patient with vulvar melanoma. After irradiation, the patient experienced mild vulvar swelling and pain, which resolved almost completely within 1 month. Four months later, the black patches gradually faded, and no local recurrence was observed 1 year after treatment. Omori et al. [156] used the BNCT to treat a patient with recurrent melanoma. The T/N and T/B ratios were measured to be 4.4 and 3.9 before treatment. After intravenous infusion of BPA, the patient received neutron irradiation at KURR in Japan. One week after the BNCT, the tumor volume substantially decreased, and no recurrence was observed within 8 months (Fig. 6E). However, 16 months after BNCT, the patient developed lung and skin metastases and eventually died of disease progression.

The Xiangya Third Hospital of Central South University reported the first case of malignant melanoma in China treated with BNCT. The patient underwent the BNCT at IHNI in Beijing on 2014 August 19. Within 6 months after the BNCT, the lesion located on the plantar aspect of the left foot had substantially regressed. Nine months after BNCT, histopathological examination of the 2 lesions revealed epidermal hyperplasia in the superficial dermis with numerous spindle-shaped pigment cells, nest formation, or invasion into the deep dermis. At 24 months post-irradiation, further regression of the lesion was noted, and no late-onset radiation-induced complications were observed during follow-up (Fig. 6B) [157].

With the continuous improvement of neutron sources, AB-BNCT has increasingly been implemented in clinical trials. Mishima was the first to apply the AB-BNCT system for the treatment of a patient with cutaneous malignant melanoma. The patient achieved PR within 6 months after treatment and CR within 12 months. During the 12-month follow-up period after BNCT, no recurrence was observed, and no treatment-related adverse events of grade 2 or higher were reported (Fig. 6C) [158,159]. Therefore, in the treatment of malignant melanoma, the BNCT has demonstrated favorable local tumor control and high response rates, particularly in cases of multiple recurrences or refractory tumors where traditional therapies are of limited efficacy. Clinical studies have shown that BNCT can effectively induce tumor regression and exhibits a favorable safety profile, with acute adverse reactions typically being mild and manageable. However, distant metastases still occur in some cases, highlighting a potential limitation of BNCT in controlling systemic disease progression. Overall, the BNCT represents a valuable local treatment strategy for patients with malignant melanoma, especially those for whom conventional treatments are ineffective or who are not surgical candidates. Future research should focus on conducting higher-quality clinical trials to further elucidate its long-term efficacy and to better define the optimal patient population.

### Clinical trial of liver cancer

**Fig. 7.**
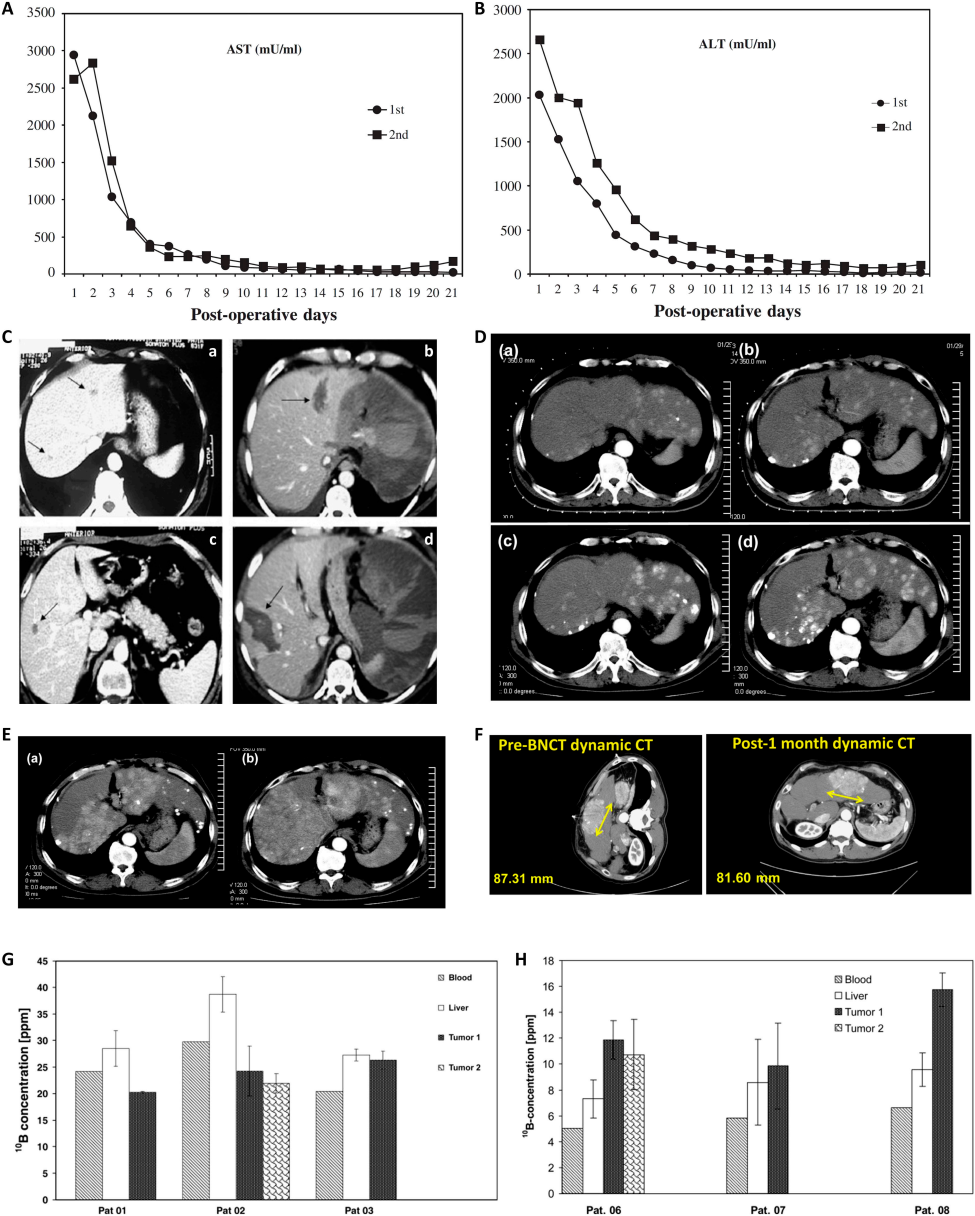
(A and B) Liver laboratory test values change in the first 3 weeks after extracorporeal BNCT in 2 patients [161]. (A) AST and (B) ALT both increased early after BNCT but decreased over 3 weeks. (C) First case of extracorporeal BNCT for metastatic hepatocellular carcinoma. The pre-BNCT CT images of the cranial (a) and caudal (c) liver aspects. CT scans on day 7 post-BNCT of the cranial (b) and caudal (d) aspects, showing necrosis surrounding the tumor sites [161]. (D) First in vivo BNCT treatment for metastatic hepatocellular carcinoma using a BSH/lipiodol emulsion as the boron delivery agent [164]. Right lobe treated with BNCT, left lobe with chemoembolization. Pretreatment CT images showed multiple tumors in both lobes (a and b); 1 month after treatment, CT images showed no growth in the right lobe but increased size in the left lobe (c and d). (E) Follow-up CT images of the first in vivo BNCT treatment for metastatic hepatocellular carcinoma [164]. (a) 3.5 months post-treatment: tumor recurrence in the right lobe. (b) 8 months post-treatment: diffuse tumor involvement in the right lobe, but no contraction or progression of BNCT-treated cirrhosis. (F) A patient with multiple hepatocellular carcinomas in the left lobe of the liver underwent BNCT after selective intra-arterial infusion of a water-in-oil-in-water emulsion containing BSH [167]. Pretreatment CT revealed multiple small hepatocellular carcinoma nodules coalescing into a large nodule (87.31 mm, left side). One-month follow-up CT showed stable tumor size (81.60 mm) with no further expansion (right side). (G and H) The uptake of BPA and BSH by different tissues was measured. BSH concentration was higher in the liver and blood than in metastatic tumors (G), while BPA concentration was higher in metastatic tumors than in the liver and blood (H). This suggests that BPA is more suitable for BNCT of metastatic liver cancer [166].

Hepatocellular carcinoma, the most prevalent form of liver cancer, represents one of the most common malignancies worldwide. Hepatectomy and liver transplantation are the main curative treatment options for patients with hepatocellular carcinoma. However, these surgical interventions require adequate liver function. For patient ineligible for surgery, alternative treatment options include percutaneous ablation, transarterial chemoembolization, radiotherapy, and systemic therapy [160]. Currently, there have also been attempts to use BNCT to treat advanced liver cancer (Table 5). Italian researcher Pinelli was the first to perform BNCT on the ex vivo liver of a patient with colorectal cancer liver metastasis and a residual liver function of 63%. The T/B ratio measured before treatment was nearly 6:1. On day 7 after BNCT, liver tissue necrosis occurred (Fig. 7C), with early increases in aspartate aminotransferase (AST) and alanine aminotransferase (ALT) (Fig. 7A and B). The patient was discharged 40 d post-BNCT with negative tumor markers and gradual recovery of residual liver function to 73%. Twenty months later, the tumor recurred, and surgical resection combined with postoperative radiochemotherapy was performed, with the patient’s disease remaining stable within 1 year. After recurrence at 33 months, conventional treatment was ineffective, and the patient eventually died 44 months post-BNCT [161,162]. In July 2003, Pinelli and colleagues [161] treated another patient with liver metastases from rectal cancer in the same way. However, due to the patient's poor cardiac function, with an ejection fraction of only 40%, their condition deteriorated 1 month after BNCT. Subsequent circulatory complications arose, including congestive heart failure. The patient eventually died after 33 d of treatment in the intensive care unit.

**Table 5.** Clinical trials of liver cancer

Author	Time	Country/region	Boron drug	Neutron source	Neutron type	Tumor type	Outcomes	Number of cases
Pinelli et al. [161,162]	2001	Italy	BPA	Triga Mark II Reactor	Epithermal	Colorectal cancer metastasis	Death at 44 months post-BNCT	1
Pinelli et al. [161]	2003	Italy	BPA	Triga Mark II Reactor	Epithermal	Colorectal cancer metastasis	Died	1
Suzuki et al. [164]	2005	Japan	BPA, BSH	KURR	Epithermal	Multifocal hepatocellular carcinoma	Died of liver failure due to tumor progression	1
Yanagie et al. [167]	2011	Japan	BSH	KURR	Epithermal	Multifocal hepatocellular carcinoma	Tumor stable for 3 months post-BNCT	1
Yanagie et al. [168]	2012	Japan	BPA	KURR	Thermal	Liver metastasis of sigmoidal colon cancer	Tumor stable for 3 months post-BNCT	1

Suzuki et al. [163] conducted a preclinical study using a BSH/lipiodol emulsion as a boron carrier. After establishing a rat liver cancer model, the emulsion was administered via intra-arterial infusion. The tumor-to-liver boron concentration ratios measured at 1, 6, and 12 h after administration demonstrated values of 4.0, 14.9, and 6.6, respectively. These findings suggest that intra-arterial infusion of the BSH/lipiodol emulsion can selectively deliver high concentrations of ^10^B to liver tumors. Subsequently, this team used this emulsion to treat the first case of multifocal hepatocellular carcinoma. During the 3.5 months following BNCT, the patient experienced transient elevations in body temperature, AST, and ALT, which normalized within 1 week. One month after BNCT, the size of the multifocal hepatocellular carcinoma in the right lobe remained stable (Fig. 7D). However, by 3.5 months, tumor regrowth in the right lobe was observed (Fig. 7E), and by 8 months, progression of right lobe liver cirrhosis was noted (Fig. 7E). The patient finally died of liver failure due to tumor progression 10 months after BNCT [164]. The team also compared the dose distribution between AB-BNCT and reactor-based BNCT for the treatment of multiple liver tumors and malignant pleural mesothelioma. The dose delivered to liver tumors by AB-BNCT was substantially higher than that by reactor-based BNCT, suggesting that AB-BNCT may be more suitable for treating tumors in the trunk region [165]. Wittig et al. [166] compared the uptake of BPA and BSH in patients with metastatic liver cancer. Among the 6 patients, 3 received BSH and the remaining 3 received BPA. The results showed that BSH concentrations in the liver and blood were higher than that in the metastases, whereas BPA concentrations were higher in the metastases than in the liver and blood. The metastasis-to-liver BSH concentration ratio was 6.8 ± 1.7, indicating that BPA is more suitable for BNCT treatment of metastatic liver cancer (Fig. 7G and H).

Japanese researcher Yanagie treated a patient with multifocal hepatocellular carcinoma in the left lobe of the liver using a water-in-oil-in-water emulsion of BSH as the boron carrier. The patient received neutron irradiation at KURR in August 2011. The size of the tumor area remained stable for 3 months after BNCT (Fig. 7F). No adverse events related to BNCT were observed during the follow-up period [167]. Additionally, Yanagie et al. [168] also reported the outcome of BNCT treatment in a male patient with sigmoid colon cancer liver metastasis in 2012. After conventional treatments, the volume of the liver metastases continued to increase, causing local pain. Evaluation of the metastatic lesions revealed that chemotherapy and molecular targeted therapy were ineffective. The patient received BNCT treatment and underwent neutron irradiation at KURR in January 2012. Follow-up abdominal CT 3 months after BNCT showed that the size of the tumor in the left lobe remained unchanged, indicating SD, and local edema was relieved. However, 7 months after BNCT, the patient died from liver failure due to metastatic lesions in the liver and respiratory failure caused by metastasis to the lungs.

In the clinical exploration of treating hepatocellular carcinoma, the BNCT has demonstrated potential application value, particularly as a novel therapeutic avenue for patients with locally advanced or refractory disease following multiple prior treatments. Existing clinical evidence indicates that BNCT can achieve effective tumor control and negative conversion of biomarkers in some patients, with an overall favorable safety profile. However, individual differences in therapeutic response are observed, with some cases still experiencing distant recurrence or disease progression. Overall, the BNCT remains in a preliminary exploratory phase for hepatocellular carcinoma. Critical issues such as developing efficacy prediction models, refining patient selection criteria, and optimizing combination therapy regimens require systematic validation through more prospective clinical studies and long-term follow-up.

### Clinical research on other types of tumors

With the continuous development of BNCT, its therapeutic scope is also expanding. In addition to the tumor types previously discussed, clinical trials have also been conducted for lung cancer, breast cancer, extramammary Paget’s disease, osteosarcoma, clear cell sarcoma, malignant peripheral nerve sheath tumor, angiosarcoma, thyroid cancer, recurrent chordoma, and gastrointestinal malignancies (Table 6).

**Table 6.** Research on other types of tumors

Author	Time	Country/region	Boron drug	Neutron source	Neutron type	Tumor type	Outcomes	Number of cases
Suzuki et al. [170]	2005–2006	Japan	BPA	KURR/JRR-4	Epithermal	Malignant pleural mesothelioma/malignant short spindle cell carcinoma	Partial regression of the tumor	1/1
Hiratsuka et al. [155]	2005–2014	Japan	BPA	KURR	Epithermal	Extramammary Paget’s disease/external genital melanoma	CR: 3 cases	3/1
Inoue et al. [178]	2008	Japan	BPA	KURR	Epithermal	Malignant peripheral nerve sheath tumor	Achieve SD	1
Makino et al. [175]	2012	Japan	BPA	KURR	Epithermal	Extramammary Paget’s disease	Complete tumor regression	2
Suzuki et al. [171]	2012	Japan	BPA	KURR	Epithermal	Recurrent lung cancer	Recurrence at the irradiation field margin	1
Yanagie et al. [168]	2011–2012	Japan	BPA	KURR	Thermal	Rectal cancer/metastatic cervical gastric tumor	SD: 2 cases; PR: 1 case	2/1
Futamura et al. [176]	2014	Japan	BPA	KURR	Epithermal	Radio-induced osteosarcoma	Tumor size decreased in 3 months	1
Pan et al. [181]	2018	Taiwan province of China	BPA	THOR	Epithermal	Recurrent papillary thyroid carcinoma	Tumor regression	1
Fujimoto et al. [177]	2019	Japan	BPA	KURR	Epithermal	Clear cell sarcoma	Complete regression	1
Igaki et al. [180]	2019–2021	Japan	Borofalan	CICS-1 (AB-BNCT)	Epithermal	Scalp angiosarcoma	CR: 2 cases	2
Kashihara et al. [158]	2019–2022	Japan	Borofalan	CICS-1 (AB-BNCT)	Epithermal	Scalp angiosarcoma/Melanoma	CR: 1 case; 1-year OS rate: 90%; PFS rate: 40%; LPFS rate: 40%	9/1
Watanabe et al. [179]	2021	Japan	BPA	THOR	Epithermal	Recurrent malignant peripheral nerve sheath tumor	Recurrence after 3 and a half months	1
Fujimoto et al. [173]	2023	Japan	BPA	KURR	Epithermal	Recurrent breast cancer	Died of lung metastasis 6 months after BNCT	1
Liao et al. [182]	2023–2024	Taiwan province of China	BPA	THOR	Epithermal	Recurrent chordoma	Tumor volume reduces	1
Kurosaki et al. [174]	2024	Japan	Borofalan	NeuCure BNCT System	Epithermal	Recurrent breast cancer	No radiation pneumonitis	3

OS: overall survival; PFS: progression-free survival; LPFS: locoregional progression-free survival

**Fig. 8.**
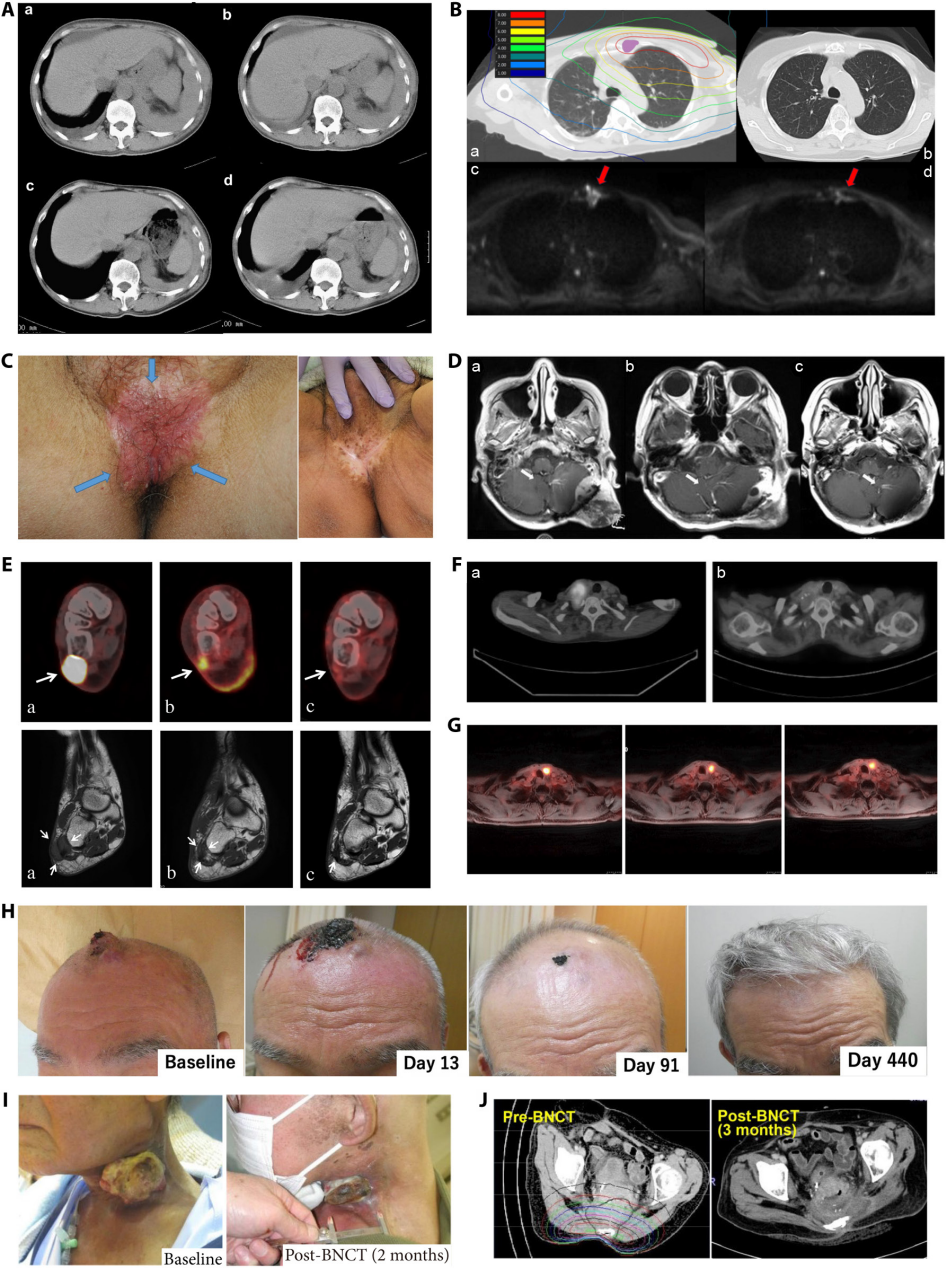
(A) CT images of a patient with malignant pleural mesothelioma are shown [170]. (a) Pre-BNCT. (b) Slight tumor regression at 1 month. (c) Partial response with tumor regression at 6 months. (d) Tumor enlargement near thoracic vertebrae at 7 months. (B) Lung dose distribution map during BNCT for recurrent breast cancer, with purple indicating gross tumor volume (a); CT scan 90 d after BNCT showed no radiation pneumonitis (b); diffusion-weighted imaging MRI revealed the tumor (red arrow) before treatment (c); MRI at 90 d after BNCT showed reduced tumor signal intensity (d) [174]. (C) Male patient with extramammary Paget’s disease [155]. Erythematous perineal lesion before BNCT (left); complete tumor regression and depigmentation after BNCT (right). (D) Gd-enhanced T1-weighted MRI of radiation-induced osteosarcoma during BNCT treatment [176]. Tumor mass visible subcutaneously and epidurally 1 month pre-BNCT (a); tumor volume reduced at 4 d after BNCT (b) and further reduced at 3 months post-BNCT (c). (E) Treatment course of clear cell sarcoma of the right foot peroneal tendon [177]. First row of pictures are ^18^F-FDG-PET/CT images. High FDG uptake (SUVmax 22.8) before first BNCT, no metastasis (a); SUVmax reduced to 3.8 after first BNCT (b); no FDG uptake after second BNCT (c). The second row of pictures are MRI T1-weighted images. Tumor visible before BNCT (a’); reduced size and signal changes 1 month after first BNCT (b’); tumor no longer visible 1 month after second BNCT (c’). (F) PET-CT of malignant peripheral nerve sheath tumor [178]. The pre-BNCT PET-CT shows high BPA uptake in tumor (a). FDG uptake normalized 18 months post-BNCT (b). (G) FDG-PET/MRI of recurrent papillary thyroid carcinoma before and after BNCT [181]. SUVmax was 17.8 pre-BNCT (left), 11.7 at 1 month after BNCT (middle), and 11.3 at 5 months post-BNCT (right). (H) Scalp angiosarcoma treatment course with BNCT [158]. (I) Pretreatment and 2-month post-treatment CT scans demonstrated growth suppression of the metastatic cervical gastric tumor [168]. (J) Pretreatment and 3-month post-treatment MR images showed stable tumor size in the patient with rectal cancer [168].

Suzuki et al. [169] conducted a fundamental experiment, by implanting squamous cell carcinoma into the thoracic cavity of mice to establish a lung cancer model, and found that the survival time of mice in the BNCT group was prolonged by 31 d compared with the control group, although the incidence of mild pulmonary fibrosis was markedly higher. Subsequently, Suzuki et al. [170] treated 2 lung cancer patients with BNCT. One patient with malignant pleural mesothelioma had a T/B ratio of 3.0 and underwent 2 BNCT treatments, 1 month apart. The patient’s chest pain resolved after the first BNCT, and CT scans at 1 and 6 months after the second BNCT confirmed PR, with no progression observed in the consolidation of the left lower lobe for 6 months (Fig. 8A). The other patient had a malignant short spindle cell tumor with a T/B ratio of 2.0 and also received 2 BNCT treatments. After the first BNCT, the symptoms of left back pain disappeared, and CT scans at 1 and 3 months showed regression of the tumor located in the lower part of the left lung; in the second round of BNCT treatment, the size of the upper lung tumor remained stable within 3 months after treatment. However, all tumors increased in size within 7 months after BNCT, and the patient eventually died of tumor progression 18 months after the first BNCT. In addition, Suzuki et al. [171] reported a case of a patient with recurrent lung cancer in the chest wall treated with BNCT in 2012. The patient had local tumor recurrence after undergoing 2 surgeries and multiple courses of radiochemotherapy, and the recurrent tumor invaded the left lower ribs. The patient underwent 2 BNCT sessions spaced 1 month apart. Follow-up fluorine-18 fluorodeoxyglucose positron emission tomography/computed tomography (^18^F-FDG-PET/CT) at 2 months after treatment showed SD, with the maximum standardized uptake value (SUVmax) of the tumor decreasing from 22.1 to 7.1. Chest pain resolved 3 months after treatment, and marked tumor regression was observed 7 months after BNCT. No severe acute or late adverse reactions occurred during the treatment process. However, local tumor recurrence was observed at the margin of the BNCT irradiation field 9 months after BNCT.

Gadan et al. [172] proposed the use of BNCT for the treatment of patients with locally recurrent HER2^+^ breast cancer, combining trastuzumab with immunoliposomes as boron carriers to target tumor cells overexpressing HER2 for the treatment of locally recurrent breast cancer. However, further research is needed on the delivery efficiency of the drug delivery system and clinical studies. Fujimoto et al. [173] reported on the BNCT treatment outcome in a patient with recurrent breast cancer and left axillary lymph node metastasis. The T/B ratio was 2.2 as shown by ^18^F-BPA PET/CT before BNCT treatment. Three months after BNCT, Gd-enhanced MRI showed substantial tumor shrinkage; the uptake of the left axillary metastatic tumor in ^18^F-FDG-PET/CT markedly decreased (SUVmax from 8.69 to 3.69). In addition, the patient’s left arm pain was alleviated, and neurological palsy symptoms improved. However, the patient died of lung metastasis 6 months after BNCT. Kurosaki et al. [174] applied the AB-BNCT to treat 3 patients with recurrent breast cancer. No radiation pneumonitis was observed within 3 months after BNCT, and the overall tumor volume decreased (Fig. 8B), preliminarily assessing the efficacy and safety of AB-BNCT in recurrent breast cancer.

Makino et al. [175] was the first to report the results of BNCT treatment for extramammary Paget’s disease. Two elderly patients, both over 70 years old, achieved CR after BNCT. During a follow-up period exceeding 1 year, neither tumor recurrence nor metastasis was observed. Hiratsuka et al. [155] described the BNCT treatment outcomes of 4 cases with genital malignancies. Three patients with extramammary Paget’s disease of the genitalia achieved CR within 6 months after BNCT. One patient died of heart disease 3.2 years after treatment, without evidence of tumor recurrence. The remaining 2 patients were alive without local or regional recurrence at 6.5 years (Fig. 8C) and 6.9 years after BNCT, respectively, indicating that BNCT can achieve local tumor control.

Futamura et al. [176] treated a patient with radiation-induced osteosarcoma at the KURR in Japan. The patient had experienced recurrence 1 year after undergoing surgery and chemotherapy and was subsequently scheduled to receive BNCT. The T/N ratio was measured at 3.8 before BNCT. Only 3 weeks after the BNCT, the patient was able to walk steadily without assistance again. No scalp radiation damage was observed except for alopecia. Three months after treatment, the tumor had further decreased in size, and the cerebellar edema had resolved (Fig. 8D). Fujimoto et al. [177] treated a patient with clear cell sarcoma of the right foot peroneal tendon using BNCT. BPA was used as the boron carrier, and the patient received 2 courses of BNCT irradiation at KURR, spaced 6 weeks apart. On the 4th day after the first BNCT, the tumor had markedly shrunk in size, and the subcutaneous hemorrhagic area had sloughed off as a dry scab. One month later, the tumor had further decreased in size, and ^18^F-FDG-PET/CT imaging revealed a marked reduction in FDG uptake, with no evidence of metastasis (Fig. 8E). One week after the second BNCT, mild edema was observed in the right foot. One month later, the edema had resolved, and the tumor had completely disappeared. One year of follow-up showed no signs of recurrence or metastasis. At 18 months post-treatment, a small, slow-growing tumor less than 1 cm in diameter was detected in the left lung. Following surgical resection, the disease remained stable, with no evidence of recurrence at other sites, suggesting that BNCT can achieve effective local control of clear cell sarcoma.

Inoue et al. [178] reported the first case of mediastinal tumor treated with BNCT. The patient was diagnosed with malignant peripheral nerve sheath tumor in the right supraclavicular fossa. After recurrence following surgical resection and failure of conventional radiochemotherapy, the patient received 2 courses of BNCT, spaced 3 weeks apart. Chest CT 1 year after BNCT showed a 25% reduction in tumor size, with SD maintained for 24 months. The ^18^F-BPA-PET at 18 months after BNCT showed no ^18^F-FDG uptake in the residual mass (Fig. 8F). No recurrence was observed 2 years after BNCT. Apart from transient dysphagia, the patient had a good quality of life. Watanabe et al. [179] reported a case of recurrent malignant peripheral nerve sheath tumor treated with BNCT. Pretreatment ^18^F-BPA-PET showed BPA accumulation in the tumor (SUVmax 4.28). After treatment, the tumor volume decreased. The ^18^F-BPA-PET at 3 months showed BPA accumulation at the edge of the tumor cavity. MRI at 3.5 months confirmed tumor recurrence.

Igaki et al. [180] conducted a phase I clinical trial to evaluate the safety and efficacy of AB-BNCT for localized scalp angiosarcoma. Two patients with scalp angiosarcoma without lymph node or distant metastasis were enrolled. Both patients achieved CR within 6 months after BNCT and remained free of tumor recurrence at 20 months after BNCT. Apart from a grade 3 asymptomatic increase in serum amylase level, no other serious treatment-related adverse events were observed during the treatment. Between November 2019 and April 2022, Kashihara et al. [158] treated 9 patients with scalp angiosarcoma and 1 patient with melanoma using CICS-1-based neutron irradiation therapy. The median follow-up period was 26.5 months. One patient with scalp angiosarcoma achieved CR (Fig. 8H). The 1-year OS rate, PFS rate, and LPFS rate were 90.0%, 40.0%, and 40.0%, respectively. The 2-year OS rate, PFS rate, and LPFS rate were 90.0%, 15.0%, and 40.0%, respectively.

Additionally, Pan et al. [181] from Taipei Veterans General Hospital treated a patient with recurrent papillary thyroid carcinoma, who had residual lesions after surgical resection, postoperative radiotherapy, and 2 courses of adjuvant radioactive iodine-131 therapy. In 2018, the patient underwent BNCT at THOR. One month after the BNCT, PET/MRI revealed regression of the recurrent tumor, with the maximum standardized boron uptake value decreasing from 17.8 to 11.7 (Fig. 8G). Serum thyroglobulin levels also decreased markedly from 55.6 ng/ml to 15.1 ng/ml. No severe adverse effects were observed during the follow-up period. Liao et al. [182] reported the first case of recurrent chordoma treated with BNCT. The patient had previously undergone 3 endonasal surgeries, one photon therapy session, and one proton therapy session, yet experienced tumor recurrence. Following evaluation, the patient received 2 courses of BNCT treatment at THOR in 2023. Two weeks after the first BNCT treatment, the patient developed mild erythema and swelling on the right cheek, as well as oral ulcers; all symptoms improved with supportive care. PET and MR images taken 2 months later revealed a reduction in tumor volume. MRI performed 1 month after the second BNCT treatment also showed further tumor shrinkage.

In gastrointestinal malignancies, the BNCT continues to show considerable therapeutic potential. Yanagie et al. [168] reported the short-term efficacy of BNCT in 3 patients with recurrent tumors after multiple surgeries and chemoradiotherapy at KURR, including 2 with rectal cancer and 1 with gastric cancer. At the 2-month follow-up after treatment, the patient with gastric cancer exhibited marked tumor burden reduction, achieving PR (Fig. 8I). The other 2 patients with rectal cancer showed SD (Fig. 8J). Unfortunately, all 3 patients died within 1 year due to tumor progression. The BNCT has shown promising therapeutic potential in several less-common malignancies such as lung carcinoma, breast cancer, and bone or soft-tissue sarcomas. Select clinical series report substantial local tumor control and symptomatic relief in patients in whom conventional modalities have failed, with an acceptable overall tolerability profile. However, current evidence remains exploratory; efficacy varies across histologies, and long-term outcomes are undefined. The expansion of BNCT applications is currently limited by boron delivery efficiency, heterogeneity in treatment protocols, and a lack of standardized patient selection criteria. Therefore, systematic prospective trials with extended follow-up are therefore imperative to establish the definitive role and criteria for BNCT in these settings.

## Perspectives and Conclusion

As a predominant contributor to global mortality, malignant neoplasms impose a dual burden of escalating economic expenditures and profound psychosocial distress across affected populations. According to statistics, the global incidence of cancer is projected to increase by 47% by 2025 [183]. Given the urgent need to improve cancer prevention, screening, diagnosis, and management strategies, there is a pressing demand for the development of effective therapeutic approaches. Currently, the main treatments for cancer include surgery, chemotherapy, and radiotherapy, with specific methods selected based on the type of tumor. However, despite the treatment modality employed, disease control remains challenging, often resulting in low long-term survival rates and suboptimal quality of life for patients. Consequently, there is a pressing need for novel therapeutic strategies to address these limitations. Emerging modalities such as immunotherapy, molecular-targeted therapy, photon therapy, proton beam therapy, carbon-ion radiotherapy, and BNCT offer promising therapeutic efficacy with reduced toxicity, thereby providing renewed hope for improved cancer management. The BNCT is an emerging targeted radiotherapeutic modality designed to improve treatment outcomes for tumors that are traditionally refractory to conventional therapies. The efficacy of the BNCT critically depends on the development of boron delivery agents capable of selectively accumulating within tumor tissues while sparing normal cells. Additionally, the choice and optimization of the neutron source substantially influence therapeutic outcomes. To date, several clinical trials have evaluated the potential of BNCT across various tumor types. However, interpretation of these findings warrants caution due to marked heterogeneity among studies, including differences in patient selection criteria, boron compounds utilized, infusion protocols, and neutron dose parameters, thereby limiting interstudy comparability. Furthermore, the small sample sizes of most trials constrain the statistical power and generalizability of the results. Notably, the majority of available evidence is derived from early-phase (phase I/II) studies, with no phase III randomized controlled trials conducted to date. Comparative analyses between BNCT and standard-of-care therapies are also lacking. These limitations underscore the need for well-designed, prospective, and adequately powered clinical trials to definitively establish the clinical utility of BNCT.

Preliminary clinical trial data suggest that BNCT yields encouraging therapeutic outcomes, supported by a growing body of preclinical evidence confirming its safety and efficacy. Advances in boron delivery agents and neutron source technologies have further enhanced the therapeutic potential of BNCT. For example, the combination of ^10^B and ^18^F facilitates the therapeutic research of BNCT. Numerous clinical trials have demonstrated that ^18^F-BPA (^18^F-labeled BPA) has been instrumental in BNCT clinical trials, contributing to patient screening, treatment planning, and efficacy evaluation. Prior to treatment, ^18^F-BPA-PET imaging noninvasively monitors tumor boron uptake and accurately measures T/N ratio, identifying patients suitable for BNCT [67,95,104]. This imaging technique also provides crucial data for BNCT treatment planning [156,174]. By reconstructing the distribution of boron carriers in the body, PET imaging helps formulate neutron irradiation doses and ranges, ensuring effective tumor irradiation while minimizing normal tissue exposure. Post-treatment, ^18^F-BPA-PET assesses BNCT efficacy by detecting reduced tumor uptake of ^18^F-BPA [85,106,178]. Given these advantages, ^18^F-BPA holds promise for future cancer treatment breakthroughs, potentially expanding its application to more cancer types, as ongoing research deepens our understanding of boron carriers and BNCT mechanisms and optimizes imaging technology.

In addition to boron compounds, non-boron compounds such as ^157^Gd have also attracted considerable attention due to their therapeutic mechanisms similar to those of BNCT. As an MRI contrast agent, ^157^Gd can effectively image tumor tissues. Moreover, its high uptake in tumor cells enables the occurrence of the ^157^Gd(n,γ)^158^Gd capture reaction during neutron irradiation, generating gamma rays, x-rays, and various low-energy electrons [40]. These low-energy electrons, which possess high LET, can induce double-strand breaks in the DNA of tumor cells, thereby achieving a cytotoxic effect at the cellular level [40,184]. However, the relatively large range of the gamma rays and x-rays produced by the capture reaction somewhat limits the selective cytotoxic effect of ^157^Gd, necessitating precise control of the drug dosage. Additionally, compared with boron-containing drugs, the broader range of radiation generated by ^157^Gd can also provide supplementary damage to the tissues surrounding BNCT. Currently, studies have explored the combined application of boron compounds and gadolinium-based agents to investigate their complementary effects. For instance, in a mouse model, the administration of gadolinium–boron nanocomposites not only enabled imaging but also prolonged the survival of the mice, indicating that the combination of the 2 might achieve theranostic integration [184].

Concurrently, the transition from reactor-based to accelerator-based neutron sources has expanded the clinical applicability of BNCT. Beyond monotherapy, the combination of BNCT with other therapeutic modalities is increasingly gaining attention, including chemotherapy, molecular targeted therapy, immunotherapy, and conventional photon radiotherapy, aiming to achieve a synergistic antitumor effect. Among various combination regimens, a particular approach is combining neutron capture with proton therapy [185,186]. BNCT, a localized radiotherapy modality characterized by high LET, offers distinct and complementary advantages for combination with immunotherapy. The clustered, high-density double-strand breaks and extensive oxidative damage induced by BNCT generate abundant, highly fragmented and relatively nuclease-resistant cytosolic DNA [187–189]; these abnormal nucleic acid species favor sustained activation of innate immune-sensing pathways [e.g., the cyclic GMP-AMP synthase–stimulator of interferon genes (cGAS–STING)], leading to type I interferon and pro-inflammatory cytokine production that promotes dendritic cell maturation, cross-presentation of tumor antigens, and priming of effector T cells [190,191]. The BNCT also frequently induces immunogenic cell death with concomitant release of damage-associated molecular patterns (DAMPs), further enhancing antigen presentation and immune activation [192,193]. At the tissue level, the BNCT achieves efficient tumor ablation within a short course, rapidly disrupting the tumor’s mechanical architecture and dense stroma, reducing interstitial pressure and cell–cell adhesion, and thereby improving immune-cell access to the tumor core—including circulating lymphocytes, tumor-infiltrating T cells, adoptively transferred chimeric antigen receptor T cells, and tumor-infiltrating lymphocytes [194,195]. Importantly, this short, intense local high-dose/high-LET particle irradiation produces less cumulative damage to surrounding infiltrating immune cells than protracted, fractionated low-LET radiotherapy, thereby better preserving local and systemic immune function and mitigating radiotherapy-associated immune exhaustion. Such preservation creates a more favorable tumor-immune microenvironment for subsequent administration of immune checkpoint inhibitors, STING agonists, or adoptive cell therapies and may amplify abscopal effect antitumor responses [193,196]. The combination of the BNCT with immunotherapy holds considerable potential to overcome immune resistance in solid tumor and to improve systemic disease control. Continued advances in the development of novel ^10^B agents with high tumor affinity, together with improvements in treatment planning, dosimetry, and translational research, are expected to enhance the efficacy and safety of BNCT-based combination regimens, thereby facilitating clinical implementation and ultimately improving survival and quality of life for patients with refractory malignancies.
